# Feasibility of multiomics tumor profiling for guiding treatment of melanoma

**DOI:** 10.1038/s41591-025-03715-6

**Published:** 2025-05-27

**Authors:** Nicola Miglino, Nora C. Toussaint, Alexander Ring, Ximena Bonilla, Marina Tusup, Benedict Gosztonyi, Tarun Mehra, Gabriele Gut, Francis Jacob, Stephane Chevrier, Kjong-Van Lehmann, Ruben Casanova, Andrea Jacobs, Sujana Sivapatham, Laura Boos, Parisa Rahimzadeh, Manuel Schuerch, Bettina Sobottka, Natalia Chicherova, Shuqing Yu, Rebekka Wegmann, Julien Mena, Emanuela S. Milani, Sandra Goetze, Cinzia Esposito, Jacobo Sarabia del Castillo, Anja L. Frei, Marta Nowak, Anja Irmisch, Jack Kuipers, Monica-Andreea Baciu-Drăgan, Pedro F. Ferreira, Franziska Singer, Anne Bertolini, Michael Prummer, Ulrike Lischetti, Tumor Profiler Consortium, Nicola Miglino, Nora C. Toussaint, Ximena Bonilla, Marina Tusup, Gabriele Gut, Francis Jacob, Kjong-Van Lehmann, Ruben Casanova, Andrea Jacobs, Sujana Sivapatham, Manuel Schuerch, Bettina Sobottka, Natalia Chicherova, Shuqing Yu, Rebekka Wegmann, Julien Mena, Emanuela S. Milani, Sandra Goetze, Cinzia Esposito, Jacobo Sarabia del Castillo, Anja L. Frei, Marta Nowak, Anja Irmisch, Jack Kuipers, Pedro F. Ferreira, Franziska Singer, Anne Bertolini, Michael Prummer, Ulrike Lischetti, Melike Ak, Faisal S. Al-Quaddoomi, Silvana I. Albert, Jonas Albinus, Ilaria Alborelli, Sonali Andani, Per-Olof Attinger, Monica-Andreea Baciu-Drăgan, Daniel Baumhoer, Beatrice Beck-Schimmer, Lara Bernasconi, Lars Bosshard, Byron Calgua, Stéphane Chevrier, Ricardo Coelho, Maya D’Costa, Esther Danenberg, Natalie R. Davidson, Stefanie Engler, Martin Erkens, Katja Eschbach, André Fedier, Joanna Ficek-Pascual, Bruno Frey, Linda Grob, Detlef Günther, Pirmin Haeuptle, Viola Heinzelmann-Schwarz, Sylvia Herter, Rene Holtackers, Tamara Huesser, Alexander Immer, Tim M. Jaeger, Alva R. James, Philip M. Jermann, André Kahles, Abdullah Kahraman, Werner Kuebler, Christian P. Kunze, Christian Kurzeder, Mitchell Levesque, Flavio C. Lombardo, Sebastian Lugert, Philipp Markolin, Martin Mehnert, Julian M. Metzler, Simone Muenst, Riccardo Murri, Charlotte K. Y. Ng, Stefan Nicolet, Monica Nunez Lopéz, Patrick GA Pedrioli, Salvatore Piscuoglio, Laurie Prélot, Natalie Rimmer, Mathilde Ritter, Christian Rommel, María L. Rosano-González, Natascha Santacroce, Ramona Schlenker, Petra C. Schwalie, Severin Schwan, Tobias Schär, Gabriela Senti, Wenguang Shao, Vipin T. Sreedharan, Stefan Stark, Daniel J. Stekhoven, Tanmay Tanna, Tinu M. Thomas, Markus Tolnay, Vinko Tosevski, Mustafa A. Tuncel, Audrey Van Drogen, Marcus Vetter, Tatjana Vlajnic, Sandra Weber, Walter P. Weber, Fabian Wendt, Norbert Wey, Mattheus HE Wildschut, Johanna Ziegler, Marc Zimmermann, Martin Zoche, Gregor Zuend, Rudolf Aebersold, Marina Bacac, Gerd Maass, Holger Moch, Michael Weller, Alexandre P. A. Theocharides, Markus G. Manz, Niko Beerenwinkel, Christian Beisel, Lucas Pelkmans, Berend Snijder, Bernd Wollscheid, Bernd Bodenmiller, Viktor H. Koelzer, Gunnar Rätsch, Reinhard Dummer, Andreas Wicki, Rudolf Aebersold, Marina Bacac, Gerd Maass, Holger Moch, Michael Weller, Alexandre P. A. Theocharides, Markus G. Manz, Niko Beerenwinkel, Christian Beisel, Lucas Pelkmans, Berend Snijder, Bernd Wollscheid, Viola Heinzelmann, Bernd Bodenmiller, Mitchell P. Levesque, Viktor H. Koelzer, Gunnar Rätsch, Reinhard Dummer, Andreas Wicki

**Affiliations:** 1 Department of Medical Oncology and Hematology, University of Zurich and University Hospital, Zurich, Switzerland; 2 NEXUS Personalized Health Technologies, ETH Zurich, Zurich, Switzerland; 3 SIB Swiss Institute of Bioinformatics, Lausanne, Switzerland; 4 Swiss Data Science Center SDSC, Zurich, Switzerland; 5 Department of Computer Science, Institute of Machine Learning, ETH Zurich, Zurich, Switzerland; 6 Department of Dermatology, University Hospital Zurich, University of Zurich, Zurich, Switzerland; 7 Department of Biomedicine, University Hospital Basel and University of Basel, Basel, Switzerland; 8 Department of Quantitative Biomedicine, University of Zurich, Zurich, Switzerland; 9 Department of Biology, RWTH Aachen, Aachen, Germany; 10 Department of Pathology and Molecular Pathology, University of Zurich and University Hospital, Zurich, Switzerland; 11 Department of Biology, Institute of Molecular Systems Biology, ETH Zurich, Zurich, Switzerland; 12 Department of Health Sciences and Technology, ETH Zurich, Zurich, Switzerland; 13 ETH PHRT Swiss Multi-Omics Center (SMOC), ETH Zurich, Zurich, Switzerland; 14 Department of Molecular Life Sciences, University of Zurich, Zurich, Switzerland; 15 Roche Pharmaceutical Research and Early Development, Roche Innovation Center, Zurich, Switzerland; 16 Department of Biosystems Science and Engineering, ETH Zurich, Basel, Switzerland; 17 Roche Diagnostics GmbH, MWG, Penzberg, Germany; 18 Department of Neurology, University Hospital and University of Zurich, Zurich, Switzerland; 19 Institute of Medical Genetics and Pathology, University Hospital Basel, Basel, Switzerland; 20 Biomedical Informatics, University Hospital Zurich, Zurich, Switzerland; 21 AI Center at ETH Zurich, ETH Zurich, Zurich, Switzerland; 22 Department of Biology, ETH Zurich, Zurich, Switzerland; 23 F. Hoffmann-La Roche Ltd, Basel, Switzerland; 24 University of Zurich, VP Medicine, Zurich, Switzerland; 25 University Hospital Zurich, Clinical Trials Center, Zurich, Switzerland; 26 University Hospital Basel, Basel, Switzerland; 27 Roche Pharmaceutical Research and Early Development, Roche Innovation Center, Basel, Switzerland; 28 Life Science Zurich Graduate School, Biomedicine PhD Program, Zurich, Switzerland; 29 ETH Zurich, Department of Chemistry and Applied Biosciences, Zurich, Switzerland; 30 Cantonal Hospital Baselland, Medical University Clinic, Liestal, Switzerland; 31 Max Planck ETH Center for Learning Systems, Zurich, Switzerland; 32 FHNW, School of Life Sciences, Institute of Chemistry and Bioanalytics, Muttenz, Switzerland; 33 University Hospital Basel, Department of Information- and Communication Technology, Basel, Switzerland; 34 University Hospital Basel, Brustzentrum, Basel, Switzerland; 35 University Hospital Zurich, Department of Gynecology, Zurich, Switzerland; 36 University of Bern, Department of BioMedical Research, Bern, Switzerland; 37 Roche Pharmaceutical Research and Early Development, Roche Innovation Center Munich, Roche Diagnostics GmbH, Penzberg, Germany; 38 University Hospital Basel, Brustzentrum and Tumorzentrum, Basel, Switzerland; 39 Department of Surgery, Brustzentrum, University Hospital Basel and University of Basel, Basel, Switzerland; 40 University Hospital Zurich, Zurich, Switzerland

**Keywords:** Melanoma, Cancer genomics, Tumour immunology, Tumour biomarkers, Cancer therapy

## Abstract

There is limited evidence supporting the feasibility of using omics and functional technologies to inform treatment decisions. Here we present results from a cohort of 116 melanoma patients in the prospective, multicentric observational Tumor Profiler (TuPro) precision oncology project. Nine independent technologies, mostly at single-cell level, were used to analyze 126 patient samples, generating up to 500 Gb of data per sample (40,000 potential markers) within 4 weeks. Among established and experimental markers, the molecular tumor board selected 54 to inform its treatment recommendations. In 75% of cases, TuPro-based data were judged to be useful in informing recommendations. Patients received either standard of care (SOC) treatments or highly individualized, polybiomarker-driven treatments (beyond SOC). The objective response rate in difficult-to-treat palliative, beyond SOC patients (*n* = 37) was 38%, with a disease control rate of 54%. Progression-free survival of patients with TuPro-informed therapy decisions was 6.04 months, (95% confidence interval, 3.75–12.06) and 5.35 months (95% confidence interval, 2.89–12.06) in ≥third therapy lines. The proof-of-concept TuPro project demonstrated the feasibility and relevance of omics-based tumor profiling to support data-guided clinical decision-making. ClinicalTrials.gov identifier: NCT06463509.

## Abstract (web summary)

In the Tumor Profiler proof-of-concept observational study, a multiomics approach for profiling tumors from patients with melanoma was feasible, returning data within 4 weeks and informing treatment recommendations in 75% of cases.

## Main

Clinical decision-making in oncology is based on data derived from clinical trials that provide standardized measures of efficacy and safety. These data are evaluated by expert panels and incorporated into clinical practice guidelines of the main medical societies, which define the evidence-based standard of care (SOC) and guide diagnostic and treatment procedures through a process of shared decision-making between physicians and patients^1,2^. Adhering to these guidelines has been shown to improve survival rates and other outcome measures in patients with cancer^3–5^. In the case of malignant melanoma, prognosis has improved dramatically over the past decades due to implementation of efficacious SOC treatments^6,7^. However, many melanoma patients experience recurrence or progression of their disease. Evidence-based treatment options for these patients are limited and clinical management presents a major challenge that often falls outside the scope of clinical guidelines.

The advent of reliable and affordable functional and omics technologies, including analyses at single-cell resolution, has rapidly transformed the field of oncology^8–11^. Clinical trials and practice guidelines are increasingly incorporating multidimensional data that enable an unprecedented characterization of molecular drivers and therapeutic vulnerabilities specific to the individual patient’s cancer, paving the way for personalized precision treatments^12,13^. Moreover, omics-based patient data can guide enrollment of patients in suitable clinical trials^14^. To integrate molecular and functional data into clinical decision-making, institutional and national multidisciplinary molecular tumor boards (MTBs) have been established^15^. Previously, we and others have reported on the development of pipelines that enable the use of molecular data by MTBs^10,16,17^. Despite the promise of precision medicine, there is limited evidence supporting feasibility and utility of using multiomics and single-cell technologies to guide selection of treatments for patients with cancer.

The Tumor Profiler (TuPro) project is a multicentric, prospective, nonrandomized observational project designed to assess the relevance of functional, single-cell and bulk omics readouts to MTB decisions^18^. The goal of the TuPro project is to inform both guideline-based decision-making for patients in the SOC setting, and data-driven decision-making to provide information on the status of additional, potentially relevant biomarkers beyond SOC. Here we present the outcomes for 116 melanoma patients enrolled in the TuPro project.

A fast diagnostic loop was implemented, enabling an overall turnaround time of 4 weeks from biopsy to reporting in the MTB with single-cell genomics (scDNA-seq)^19^ and transcriptomics (scRNA-seq)^20^, targeted proteomics (imaging mass cytometry (IMC)^21^, cytometry by time of flight (CyTOF)^22^), proteotyping (using data-independent acquisition (DIA)^23–25^), drug phenotyping (Pharmacoscopy^11,26^, iterative indirect immunofluorescence imaging (4iDRP)^27^), targeted next-generation DNA sequencing (NGS) and digital pathology (DigiPath). The selection of these technologies was based on their capability to provide a comprehensive molecular portrait of each tumor with potential clinical relevance. As a result, up to 500 Gb of data were generated and evaluated for each sample, corresponding to over 40,000 potential markers for therapy decisions. The molecular data were integrated with additional clinical and diagnostic information available for each patient during the MTB evaluation. Markers used for treatment recommendations, administered therapies, objective responses and outcomes were captured and analyzed within the project framework.

## Results

### Patient cohort and sample characteristics

**Fig. 1:**
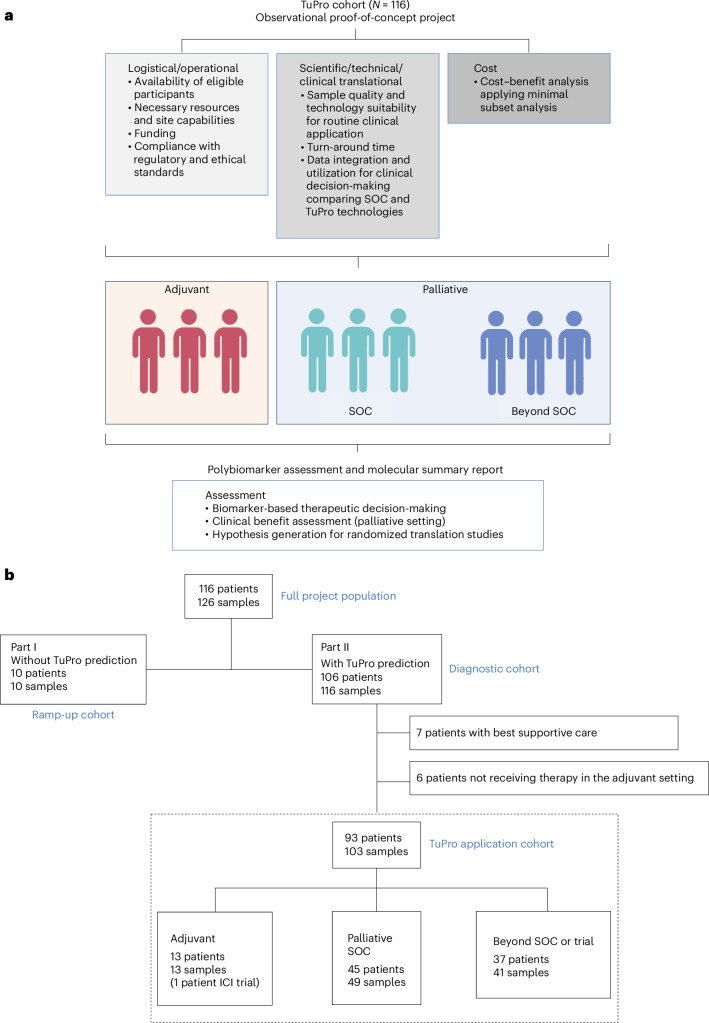
Project overview. **a**, Project description showing principal aspects for feasibility testing, patient populations included and information utilization. **b**, CONSORT flow diagram of the TuPro project. A total of 116 patients were included in the TuPro melanoma project. Part I included ten patients to establish the TuPro workflow and analysis/reporting pipeline. Part II included the remaining 106 patients in the diagnostic cohort. Thirteen patients either received BSC (*n* = 7) or did not receive adjuvant treatment based on shared decision-making (*n* = 6). The remaining 93 patients formed the TuPro application cohort, which was categorized into three groups: (1) adjuvant therapy, (2) SOC palliative therapy and (3) palliative therapy beyond SOC. In both palliative SOC and beyond SOC groups, more than one sample was evaluated for four patients. Two additional patients are both in the SOC and beyond SOC at different treatment lines. Illustrations in **a** created using BioRender.com.

A total of 116 patients with any subtype of melanoma participated in the project, contributing 126 biopsies (including ten longitudinal samples) (Fig. 1). The enrollment period spanned 21 months from January 2019 to November 2020. The demographical, clinical and pathological features of eligible patients were recorded in a good-clinical-practice-compliant database (Extended Data Table 1). Among all biopsy specimens, 62% were obtained from patients who had been treated with at least one therapeutic regime; 43% of these patients had undergone two or more lines of treatment (Extended Data Table 1). Biopsies from ten patients were used for the project ramp-up phase, whereas 106 underwent the analysis and the discussion at the MTB (Fig. 1). Thirteen patients were excluded from further analysis; of those, seven transitioned to best supportive care (BSC) due to deteriorating clinical condition and six early-stage patients opted against the recommended therapy in the adjuvant setting. The remaining 93 patients (103 biopsy samples, 99 treatment lines) were subject to the analysis in this paper and correspond to the TuPro application population. Cases were discussed at the MTB, including guideline-based treatment options, as well as molecular and functional profiles obtained through tumor profiling, according to three patient groups:

Group 1—adjuvant setting: 13 patients with early-stage disease who underwent adjuvant treatment. These patients were administered targeted tyrosine kinase inhibitor (TKI) therapy for BRAF-mutant tumors or immunotherapy involving anti-PD1 agents. One patient received anti-CTLA-4 and anti-PD1 antibodies in the framework of an interventional trial.

Group 2—palliative SOC: 45 patients with metastatic disease who received palliative SOC therapy. These treatment regimens included immunotherapy, targeted therapy, talimogene laherparepvec and chemotherapy.

Group 3—palliative beyond SOC (henceforth referred to as beyond SOC): 37 patients with metastatic disease who underwent palliative treatments beyond SOC. These interventions comprised participation in interventional trials during any time of disease course, or the application of individualized treatments (that is, off-label drug use, or the off-label reintroduction of approved therapies following confirmed progression upon initial treatment with these agents).

The median follow-up time was 20.5 months (range 0.6–46.5 months). As of 31 May 2023, 62.4% (58 of 93) of the patients had died due to melanoma or nonmelanoma related events.

### TuPro fast diagnostic loop and technology node contributions

**Fig. 2:**
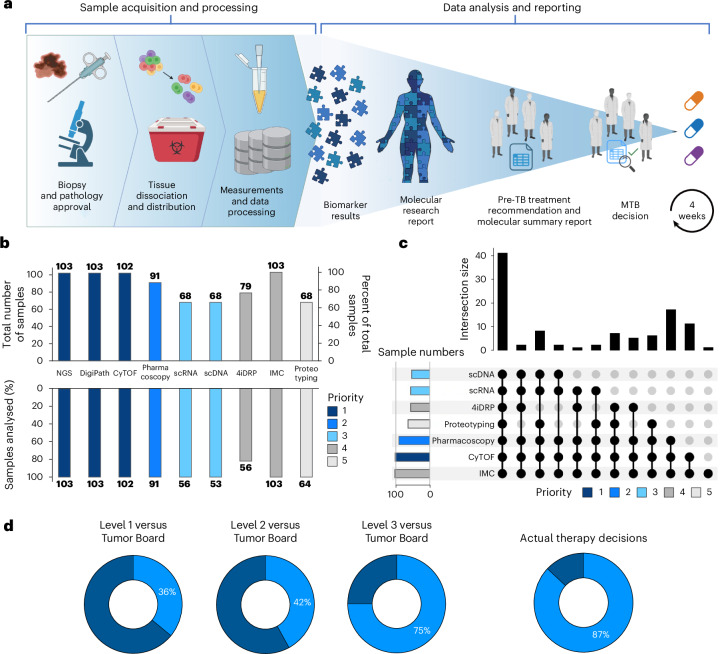
TuPro workflow, technology node performance and output. **a**, The TuPro workflow consists of a sample acquisition and processing phase, followed by an analysis and reporting phase, with a turnaround time of ≤4 weeks. The analysis phase involved nine technological nodes, although not all samples were analyzed by each node. Data processing integrated the outputs from different technologies and enabled the generation of the MSR, which encapsulates the essential findings and actionable insights for clinicians and researchers. **b**, Number of samples (*n* = 103) analyzed per technology node (*n* = 9) within the fast diagnostic loop (4-week turnaround time). Given limited sample material in some cases, the assays were prioritized as indicated by color. **c**, UpSet plot showing the number of samples analyzed by one or more technology nodes (*n* = 9). **d**, Concordance (percentage in light blue) between the recommendations agreed upon by the MTB and those inferred from information provided by diagnostic levels level 1 (detailed clinical data, routine molecular testing data in melanoma (*BRAF*, *NRAS* and *c-KIT* mutations) and DigiPath data), level 2 (level 1 plus large panel NGS), or level 3 (all TuPro technology nodes, *n* = 9), and fraction of actual TuPro-driven therapy decisions (i.e., ≥50% of the applied drugs are supported by markers measured by TuPro). Illustrations in **a** created using BioRender.com.

The successful integration of high-throughput analytical technologies into routine clinical practice requires a turnaround time that aligns with clinical decision-making. To meet this need, the TuPro project established a fast diagnostic loop workflow (Fig. 2a). The workflow mandates that each technology platform delivers a comprehensive sample-specific report for discussion at the pre-tumor board (pre-TB, see below) and subsequently at the MTB, which takes place within 4 weeks of biopsy. For each sample, data were generated and analyzed through a previously described process and information technology pipeline developed specifically for the TuPro project^18^. The data were discussed by a pre-TB expert panel and translated into marker-based treatment recommendations that were summarized in a molecular summary report (MSR). The MSR was communicated to the MTB, along with the supporting clinical and molecular data (Fig. 2a). The MTB then provided a formal treatment recommendation for the patient. Due to limitations of fresh tissue sample abundance or quality, predefined sample access priorities were assigned to individual technologies and sample processing within clinical routine practice (Fig. 2b,c). Technologies performing measurements on formalin-fixed paraffin-embedded tissue prepared for routine diagnostics (for example, IMC, NGS), were generally unaffected by limited sample abundance. IMC, targeted NGS and DigiPath received the most samples (*n* = 103), followed by CyTOF (*n* = 102), Pharmacoscopy (*n* = 91) and 4iDRP, (*n* = 79). scRNA-seq, scDNA-seq and proteotyping each received 68 samples. Most technology nodes successfully analyzed and reported results on the received samples if they passed node-specific quality requirements (Fig. 2b). Out of all samples (*n* = 103), 41 were analyzed successfully by all nine nodes (Fig. 2c). Considering each datapoint, over 40,000 measurements were assessed per sample as potential markers, of which 544 were analyzed using targeted technologies (Supplementary Table M1). Overall, the TuPro framework successfully established a robust multiomics and functional analysis pipeline that yielded rich individual tumor profiles, providing additional and timely insights for clinical decision-making.

### Pre-TB- and MTB-derived treatment recommendations

Marker data provided in the MSR was condensed into diagnostic levels that build on each other to facilitate accessibility (Methods): level 1 incorporated detailed clinical data, routine molecular testing data plus routine pathology and DigiPath data. Level 2 additionally included results of a broad targeted NGS panel. Level 3 included level 2 data, and data provided by the seven TuPro technology nodes. Final MTB recommendations were in line with data provided by levels 1, 2 and 3 in 36%, 42% and 75% of samples, respectively. The lower percentage of MTB agreement with level 1 and 2 results mainly from a frequent lack of identifying new options for targeted therapy with standard technologies. The percentage of TuPro-driven actual treatments administered to patients (that is, treatments decided by patients and their physicians after MTB recommendations, with ≥50% of the applied drugs supported by markers measured by TuPro) reached 87% (Fig. 2d). In summary, whereas the addition of extended targeted NGS (level 2) to standard clinical workup provided only a modest impact to the MTB, functional and multiomics TuPro data (level 3) provided substantial added value to clinical decision-making compared to current diagnostic standards (NGS and DigiPath). This is reflected by the increased concordance of TuPro-informed recommendations with those of the MTB by 39% (compared to diagnostic level 1) and 33% (compared to diagnostic level 2) of cases, respectively (Fig. 2d).

### Marker selection for treatment decision

**Fig. 3:**
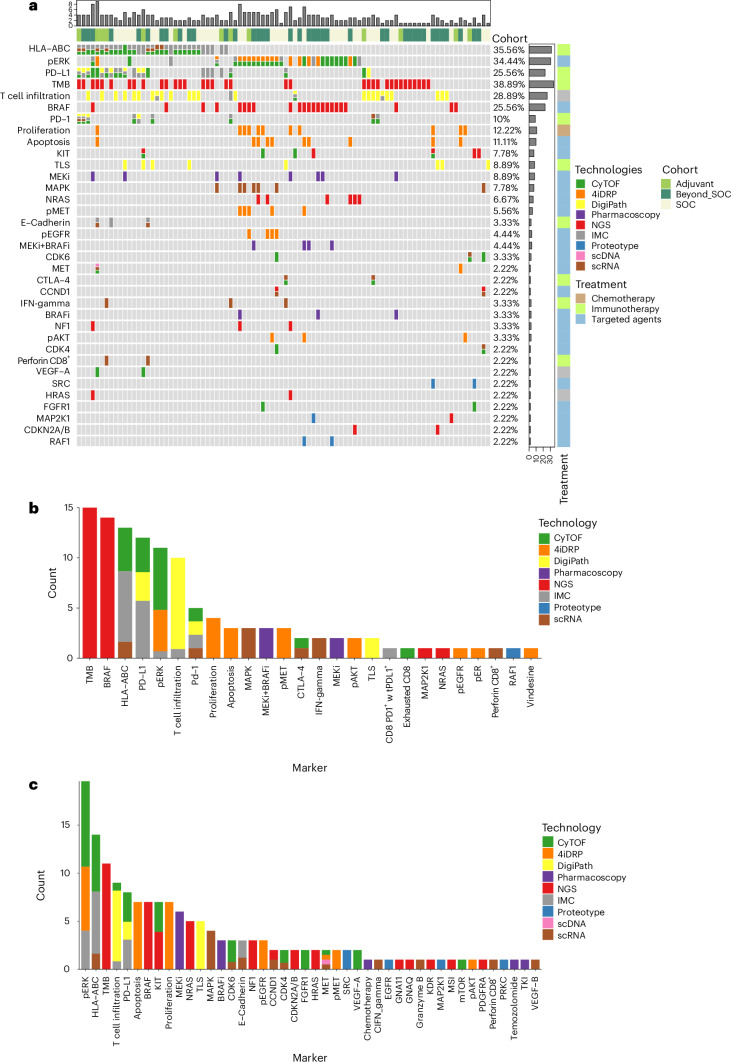
TuPro marker subset utilized in clinical decision-making. **a**, OncoPrint showing biomarkers (cutoff of >2%) detected by TuPro and used for treatment decisions (*n* = 90). Data on the technology node used to measure each marker, the cohort and class of treatment received (chemotherapy, targeted therapy, immunotherapy) are provided. **b**, Markers (*n* = 26) used for treatment decision-making in the palliative SOC cohort. **c**, Markers (*n* = 44) used for treatment decision-making in the beyond SOC cohort.

**Extended Data Fig. 1:**
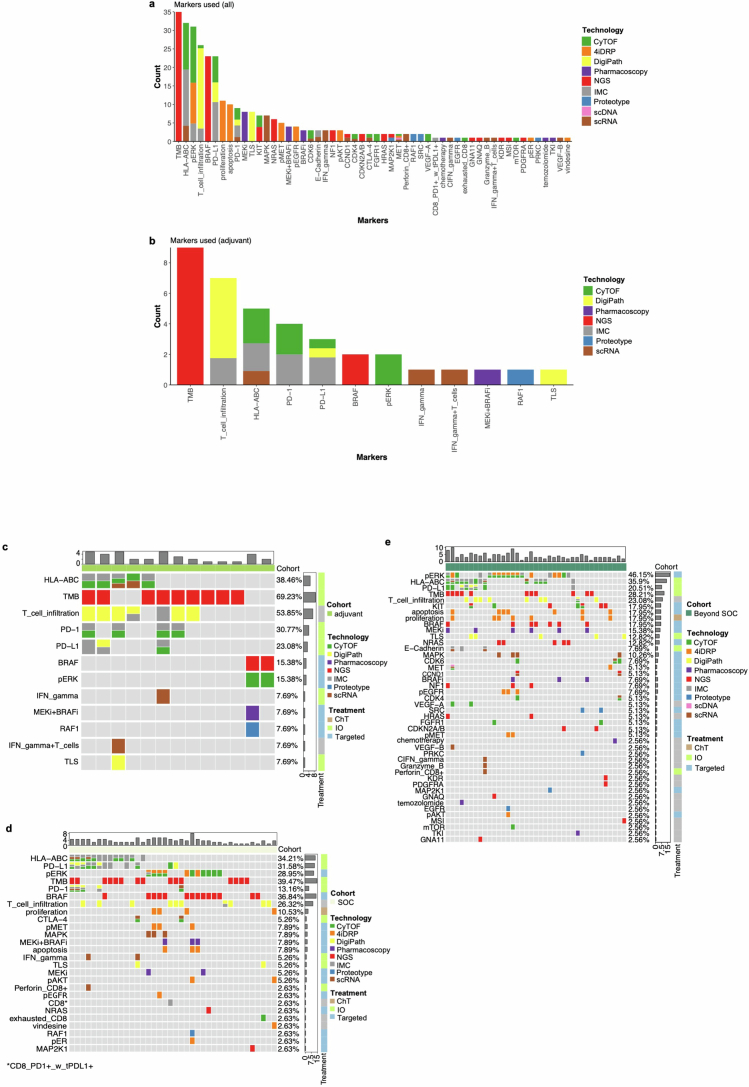
Markers used for applied therapeutic decisions. **a**, Markers (n = 54) used for treatment decision making in the adjuvant, SOC and beyond SOC cohorts. **b**, Markers (n = 12) used for treatment decision making in the adjuvant cohort. **c**, Individual measurements of markers for applied therapeutic decisions in the adjuvant setting. Markers (n = 12) and measurements (n = 50). **d**, Individual measurements of markers used for applied therapeutic decisions in the palliative SOC setting. Markers (n = 26) and measurements (n = 153). **e**, Individual measurements of markers used for applied therapeutic decisions in the beyond SOC setting. Markers (n = 44) and measurements (n = 196).

Out of all available measurements, the pre-TB and the MTB participants decided on those deemed to be most useful to inform decisions. For actual treatment decisions (that is, treatments prescribed by the treating physician and supported by TuPro measurements), a total of 54 individual markers (recorded as part of the pre-TB discussions) were used (Fig. 3a and Extended Data Fig. 1a). These 54 markers correspond to 399 individual, validated measurements, including assessment of the same target by different technologies (for example, pERK level was measured by IMC, CyTOF and 4iDRP). The distribution of individual measurements that informed treatment decisions was as follows: adjuvant setting, 50 marker measurements in 13 of 13 samples (Extended Data Fig. 1b,c); palliative SOC group, 153 marker measurements in 38 of 49 samples (Fig. 3b and Extended Data Fig. 1d) and beyond SOC group, 196 marker measurements in 39 of 41 samples (Fig. 3c and Extended Data Fig. 1e). For the remaining 13 samples, no treatments driven by markers measured with TuPro technologies were administered to the patients. The average number of markers used for individual TuPro-based treatment recommendations was four in both the adjuvant and the palliative SOC cohort, and five in the beyond SOC cohort. Markers used for decision-making in the MTB with a cutoff at >2% amongst all patients, along with the specific technologies used for their assessment, are shown in Fig. 3a. The markers that most often informed the TuPro recommendations in the adjuvant setting (*n* = 13) were TMB (*n* = 9, 69%), T cell infiltration (*n* = 7, 54%), HLA-ABC (*n* = 5, 38%) and PD-L1 (*n* = 3, 23%) (Extended Data Fig. 1b). In the palliative SOC group (*n* = 39), the markers adopted most frequently were TMB (*n* = 15, 38%), *BRAF* mutations (*n* = 14 samples, 36%), HLA-ABC (*n* = 13, 33%), PD-L1 (*n* = 12, 31%), pERK (*n* = 11, 28%), T cell infiltration (*n* = 10, 26%), PD1 expression (*n* = 5, 13%) and proliferation (*n* = 4, 10%) (Fig. 3b). In the beyond SOC group (*n* = 39) they were pERK (*n* = 18, 46%), HLA-ABC (*n* = 14, 36%), TMB (*n* = 11, 28%), T cell infiltration (*n* = 9, 23%), PD-L1 (*n* = 8, 21%), proliferation, apoptosis, *BRAF* and *KIT* alterations (*n* = 7, 18% each) (Fig. 3c). Whereas DNA alterations (for example, TMB, mutations in *RAS*, *KIT* and *BRAF*) included United States Food and Drug Administration-recognized biomarkers (according to OncoKB^12^) measurable via level 2 diagnostics, the TuPro diagnostic level 3 provided markers additional to genetic markers (for example, phosphorylation, expression and drug response). Together, these findings show that multiomics marker data generated via TuPro technologies were readily accepted and implemented by the study physicians. In all three groups, both established as well as experimental markers were considered for clinical decision-making.

**Extended Data Fig. 2:**
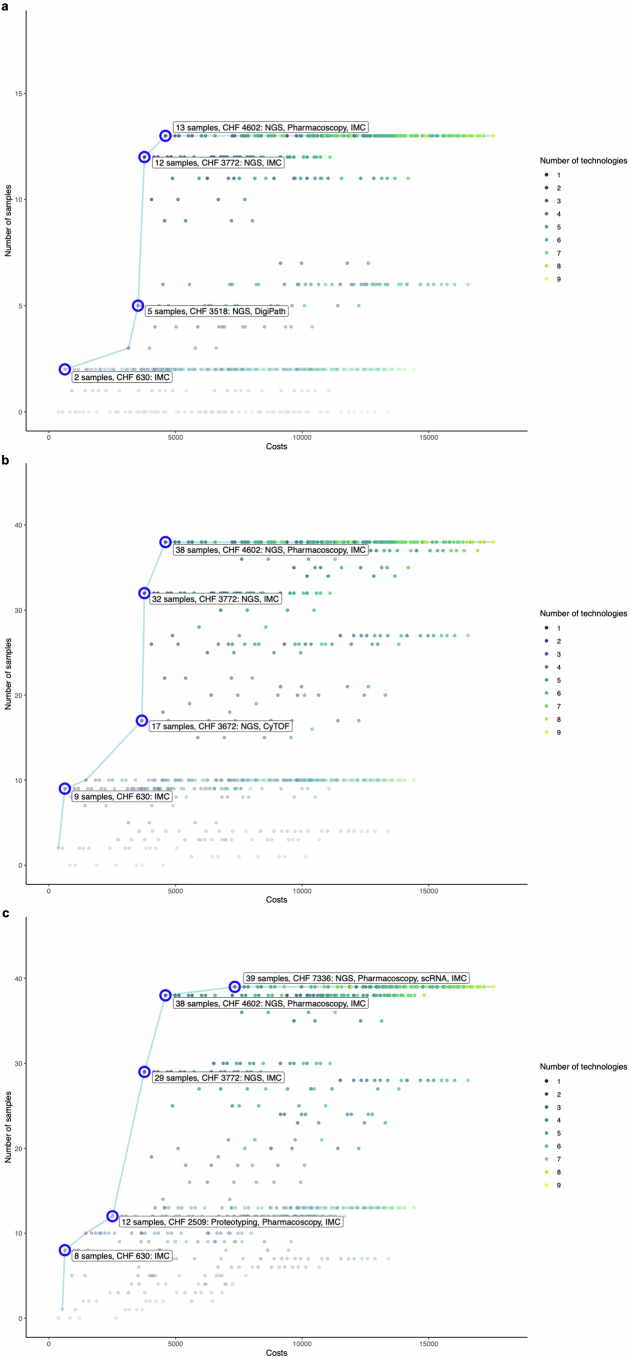
Cost analysis of technology nodes and node combinations used in the TuPro project per cohort. **a**, adjuvant, **b**, palliative SOC cohort, **c**, palliative beyond SOC. The x-axis shows the marginal costs in Swiss francs (CHF) of production, representing the cost of one additional analysis using a combination of technology nodes running at full capacity. The y-axis shows the number of patients that would have all relevant information available with the corresponding subset of technologies when compared to using all technologies. The labels on each data node indicate the individual technology nodes and combined cost per sample. Each dot corresponds to a different combination of technologies associated with a total cost for that combination. Only the best combinations are shown, as other combinations have a higher cost or a smaller number of samples with available relevant information. This analysis provides a better understanding of the cost-effectiveness of various diagnostic approaches and the trade-offs between the generated information and associated expenses.

An important question was whether similar results can be achieved with a smaller subset of technologies and markers, resulting in significantly lower profiling costs. In many instances, one marker can be measured by several technologies. A small subset of these technologies could cover all markers relevant to most patients. We performed an analysis considering all possible subsets of technologies, taking into account assumed equivalences between markers from different technologies (Supplementary Table M2a–c). For each patient, we recorded markers deemed relevant for treatment recommendations per cohort (adjuvant, SOC, beyond SOC), assuming the same recommendations would be made if all relevant markers were available. Extended Data Fig. 2 shows the number of samples (*y* axis) that had all relevant markers available for a specific combination of technologies. The *x* axis shows the cumulative cost for the combination of technologies. A cost-effectiveness analysis was performed considering all possible subsets of technologies and the fraction of patients likely to have had the same treatment recommendation (Extended Data Fig. 2a–c). Of note, only Pharmacoscopy and 4iDRP provide information for several treatment conditions, whereas the other technologies provide information only for untreated samples. We found that information provided by three technologies (NGS, Pharmacoscopy, IMC) (adjuvant and palliative SOC) and four technologies (NGS, Pharmacoscopy, IMC, scRNA-seq) (beyond SOC) would cover all markers used for pre-TB treatment recommendation (Extended Data Fig. 2a–c). This would reduce the cost per samples to 4,602 Swiss francs in the adjuvant and palliative SOC cohort, or 7,336 Swiss francs in the beyond SOC. These costs are 1.15-fold higher than the clinical-setting costs for standard NGS analysis (up to 4,000 Swiss francs in Switzerland) for adjuvant or palliative SOC, and 1.8-fold higher for beyond SOC setting.

### Treatments and outcomes in the TuPro application cohort

**Fig. 4:**
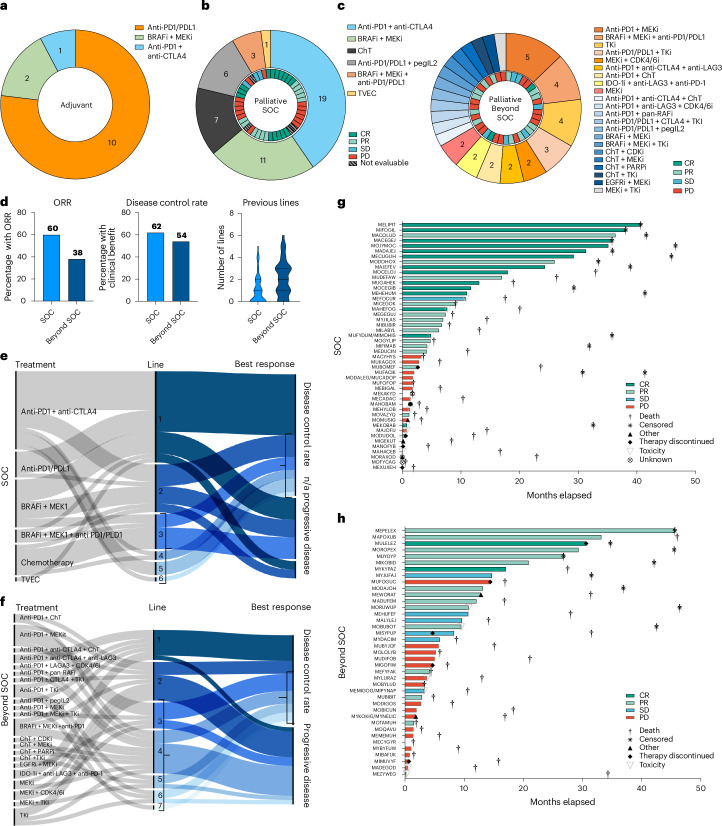
Treatment and clinical outcome parameters for the TuPro application cohort. **a**–**c**, Treatments applied in the adjuvant (*n* = 13) (**a**), palliative SOC (*n* = 47) (**b**) and beyond SOC (*n* = 39) (**c**) groups. Outer circle, therapies received; inner circle, best responses per patient categorized as CR (dark green), PR (light green), SD (light blue), PD (red) or not evaluable. **d**, ORR, disease control rate and number of previous treatment lines before biopsy for patients in the palliative SOC and beyond SOC groups. Violin plots: solid lines, median; dotted lines, first (upper) and third (lower) quartiles. **e**,**f**, Alluvial plots showing the association between types of treatment received, line in which the treatment was received and disease control rate for the palliative SOC (**e**) and beyond SOC (**f**) groups. Brackets indicate at three or more treatment lines and disease control rate. **g**,**h**, Swimmers plots showing PFS and best response to treatment for palliative SOC (**g**) and beyond SOC (**h**) groups. Two patients serially received two different treatments; another two patients received serial samplings resulting in the same treatments.

During the project period, approximately 180 Swissmedic-approved anti-cancer agents and 20 investigational drugs within 25 active trials in the participating centers were available. The TuPro workflow generated 510 treatment recommendations (including several recommendations per patient) encompassing 76 individual drugs. In the adjuvant group, 13 of 13 patients (100%) received treatment supported by markers measured with TuPro technologies. Three different systemic therapy regimens were administered (Fig. 4a). After a median observation time of 37.7 months, eight patients remained in remission and five patients had relapsed. In the palliative SOC group, 45 patients received 47 treatments considered SOC (two patients were serially biopsied and received two different treatments) (Fig. 4b). In 38 of 49 samples analyzed (77.5%), patients received a treatment supported by markers measured with TuPro technologies, and six different approved systemic treatment regimens were administered (Fig. 4b). The top three recommended treatment options were: (1) combination immune checkpoint inhibitors (ICI) (anti-CTLA-4 and anti-PD1 antibodies), (2) TKI combination therapy (BRAF/MEK inhibitors) and (3) chemotherapy (Fig. 4b)^1^. In the beyond SOC group, 37 patients received 39 treatments (two patients received serial samplings and two sequential but different treatment recommendations), either through participation in an interventional clinical trial or through off-label use of approved oncological drugs. In 39 of 41 samples analyzed, (95%) treatment decisions were informed by TuPro (Fig. 4c and Table 1). Recommendations were highly individualized and 22 different therapy regimens were used for patients in the beyond SOC group. Excluding treatment recommendations based exclusively on SOC diagnostics, TuPro level 3 data informed clinical decision-making in 9 of 13 samples from patients in the adjuvant (69.2%), 30 of 49 samples of patients in the palliative SOC (61.2%) and 35 of 41 samples of patients in the palliative beyond SOC setting (85.3%).

**Table 1:** Patients in beyond SOC group (*n* = 37) for which treatment decision was informed by TuPro

Sample	≥50% TuPro suggestions followed	Treatment after TuPro analysis	Best response	Markers used
MALYLEJ	Yes	Nivolumab + Cabozantinib	SD	tmb^1^, pERK^4^, apoptosis^4^, proliferation^4^, MET^5,7^, VEGF-A^5^, HLA-ABC^5,8^, PD-L1^5^, E-Cadherine^8^, MET^6^, VEGF-B^6^, HLA-ABC^6^, E-Cadherin^6^
MEFYFAK	Yes	Binimetinib + Encorafenib	PR	pERK^5,8^, immune desert^1^
MIMUVYF	Yes	Cobimetinib + Regorafenib	PD	GNAQ^1^, pERK^4,5^, KIT^5^, FGFR1^5^, proliferation^4^
MUBYJOF	Yes	Nivolumab + Trametinib	PD	pERK^5,8^
MYKOKIG/MYNELIC	Yes	Trametinib + Ribociclib	PD	CCND^1^ (1+2), CCND1^6^ (1+2), CDK4^6^ (2), pERGF^4^ (1), pERK^4,5^ (1), CDK4^5^ (1+2), CDK6^5^ (1+2), MAPK_pathway^6^ (1+2)
MIJYDYP	Yes	Nivolumab + Encorafenib + Binimetinib	PR	tmb^1^, BRAF^1^, HLA-ABC^5,8^
MYDACIM	Yes	Binimetinib + Pembrolizumab	SD	GNA11^1^, HLA-ABC^8^, pERK^8^, inflamed^1^
MUFOGUC	Yes	Nivolumab + Temozolomid	PD	tmb^1^, PD-L1^8^, HLA-ABC^8^
MOBICUN	Yes	Olaparib + Temozolomid	PD	MET^4^, apoptosis^4^, proliferation^4^
MECYGYR	Yes	Cobimetinib	PD	NRAS^1^, MAPK^6^, pERK^4,5^, apoptosis^4^, proliferation^4^
MYKYPAZ	Yes	Binimetinib + Encorafenib + Nivolumab	CR	tmb^1^, HLA-ABC^5,8^, pERK^5^
MULELEZ	Yes	IDO-1i + Relatlimab + Nivolumab	CR	inflamed^2^, HLA-ABC^5,8^
MEHUFEF	Yes	Nilotinib	SD	KIT^1^, apoptosis^4^, proliferation^4^, KIT^5^, SRC^9^, PRKC^9^
MYBYFUW	Yes	Imatinib	PD	imantinib^3^, KIT^5^, immune_excluded^2,5,8^
MYJUFAJ	Yes	IDO-1i + Relatlimab + Nivolumab	SD	inflamed^2^, PD-L1^5,8^ HLA-ABC^5,8^
MADUFEM	Yes	Sunitinib + Atezolizumab	PR	KIT^1^, tmb^1^, KIT^5^, VEGF^5^, TLS^2^, PD-L1^2,8^, HLA- ABC^8^
MUBIBIT	Yes	Trametinib + Ribociclib	PR	NRAS^1^, CDKN2A/B^1^, pERK^5^
MYLURAZ	Yes	Pembrolizumab + Trametinib	PD	NRAS^1^, pEKR^8^
MODAJOH	Yes	Ipilimumab + Nivolumab + Temozolomid	PR	tmb^1^, PD-L1^2,8^, HLA-ABC^5,8^, E-Cadherin^8^, temozolomide^3^
MEPELEX	Yes	PDR001 + LXH254	PR	tmb^1^
MEWORAT	Yes	Carboplatin + Sunitinib + Temozolomide	PR	KIT^1^, KDR^1^, PDGFRA^1^
MIGOFIW	Yes	Trametinib	PD	NRAS^1^, apoptosis^4^, pERK^4^
MISYPUP	Yes	Ipilimumab + Nivolumab + Relatlimab	SD	tmb, HLA-ABC^5,6^, inflammation^2^
MOBUBOT	Yes	Carboplatin + Trametinib	PR	NRAS^1^, pERK^4,5^, apoptosis^4^, pEGFR^4^
MOBYLUD	Yes	Relatlimab + Nivolumab + Ribociclib	PD	CDKN2A/2B^1^, inflammation^2^, TLS^2^
MOLOLYB	Yes	Cobimetinib + Erlotinib	PD	pERK^4,5^, pEGFR^4^, pMET^4^, pAKT^4^, apoptosis^4^, EGFR^9^
MOROPEX	Yes	Nivolumab + Binimetinib	PR	inflamed^2^, PD-L1^2,5,8^, TLS^2^, HLA-ABC^5^, MEKi^3^
MORUWUP	Yes	Ipilimumab + Nivolumab + Relatlimab	PR	MSI^1^, TLS^2^
MOQAVIJ	Yes	Dabrafenib + Trametinib + Pembrolizumab	PD	BRAF^1^, tmb^1^, MEKi^3^, BRAFi^3^
MOTAMUH	Yes	Dabrafenib + Trametinib + Regorafenib	PR	BRAF^1^, NF1^1^, pERK^4,5^, pMET^4^, proliferation^4^, mTOR^5^, MEKi^3^, BRAFi^3^, MAPK_pathway^6^
MIKOBID	Yes	Lenvatinib + Pembrolizumab or Pembrolizumab + Placebo	PR	HLA-ABC^5,8^, TLS^2^, inflamed^2^
MIBAFUK	Yes	Regorafenib	PD	BRAF^1^, KIT^1^, pERK^8^, MAP2K^9^
MADEGOD	Yes	Dacarbazin + Ribociclib	PD	dacarbazin^3^, CDK6^5^, CDK6^3^
MEZYWEG	Yes	Dasatinib	PD	KIT^1^, FGFR1^5^, SRC^9^
MIPYNAP / MEMIGOG	Yes	Nivolumab + Trametinib	SD	Tmb^1^ (1+2), BRAF^1^ (1+2), HRAS^1^ (1+2), NF1^1^ (1+2), PD-L1^5^ (2), trametinib^3^ (1+2), pERK^4^ (1), pERK^5,8^ (2), proliferation^4^ (1), HLA-ABC^5,8^ (2)
MEMEMUH	Yes	Nivolumab + PEG-IL2	PD	PD-L1^5^, HLA-ABC^5, 6, 8^, E-Cadherin^8^, E-Cadherin^6^, CIFN_gamma_CD8, Granzyme_BC_D8, Perforin_CD8
MAPOXUB	Yes	Dabrafenib + Trametinib + Spartalizumab	PR	BRAF^1^, tramentinib^3^, dabrafenib^3^, pERK^4^

Best response (CR, PR, SD), and associated markers measured by up to nine TuPro project technologies (superscript numbers): NGS^1^, digipath^2^, Pharmacoscopy^3^, 4iDRP^4^, CyTOF^5^, scRNA^6^, scDNA^7^, IMC^8^ and Proteotype^9^. (1) and (2), patients with two biopsies leading to the same treatment.

**Extended Data Fig. 3:**
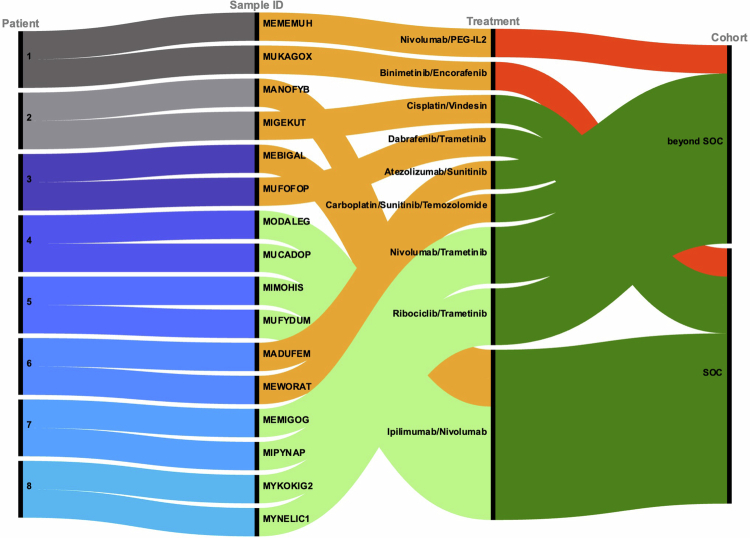
Alluvial plot for clinical implications of serial biopsies. First column (Patient) shows patients with serial biopsies (n = 8, two biopsies per patient). Second (Sample ID) and third (Treatment) column showing both biopsy samples per patient. Orange: serial biopsies leading to different treatment recommendations, light green: serial biopsies not changing treatment decision. Forth column (cohort) showing serial and biopsy decisions leading to change of classification based on clinical criteria (dark red, either palliative SOC or beyond SOC) or no change in cohort classification (dark green).

Ten patients had serial biopsies (two per patient). Two of these patients did not receive TuPro-informed recommendation and were excluded from the analysis (Supplementary Table M3). Of the remaining eight patients, two received different treatment recommendation in the SOC cohort (patient 2 and 3), one in the beyond SOC cohort (patient 6) and one patient received different recommendations leading to a switch in cohort classification (patient 1) (Supplementary Table M3 and Extended Data Fig. 3). In summary, a total of four out of eight (50%) patients with serial biopsies included in the analysis received different treatment recommendations.

For patients in the palliative SOC group, the objective response rate (ORR) was 60% and the disease control rate, which included those with complete response (CR), partial response (PR) or stable disease (SD), was 62% (Fig. 4d). The median number of previous therapy lines in the palliative SOC group was one, ranging from zero to five; in the beyond SOC group, it was two, ranging from zero to six (Fig. 4d). With a median follow-up of 17.5 months, the median duration of response was 6.5 months, ranging from 0.6 to 46.5 months. For patients in the beyond SOC group, the ORR was 38% and the disease control rate was 54% (Fig. 4d). With a median follow-up period of 16.9 months, the median duration of response was 4.8 months, ranging from 0.2 to 46.4 months (Fig. 4g,h). Analysis of disease control rate for each treatment showed that both palliative SOC and beyond SOC groups benefited from treatments administered in third and further lines (Fig. 4e,f) (brackets indicate at least three lines of treatment and disease control rate).

### Clinical benefit in TuPro compared to non-TuPro patients

**Fig. 5:**
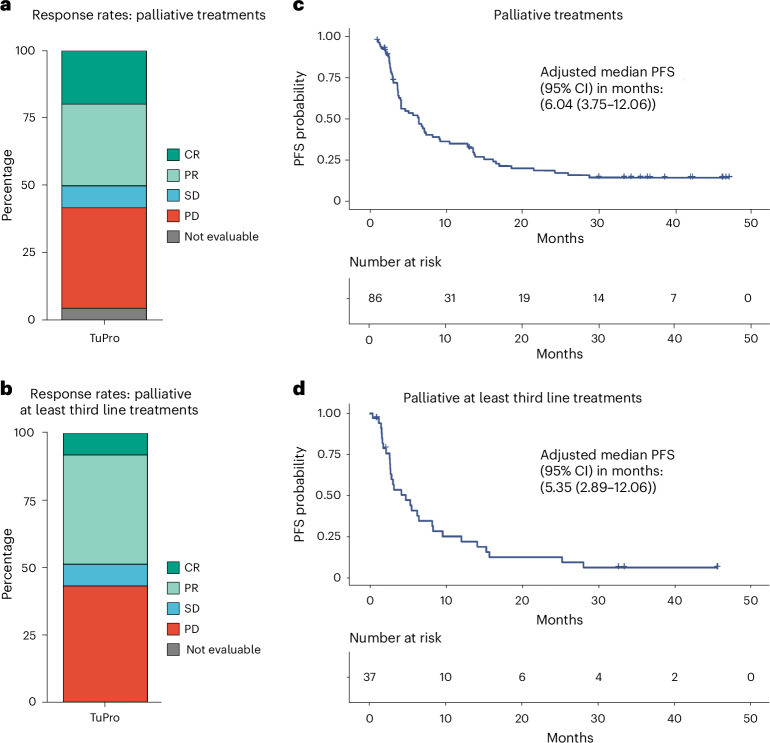
Response rates and PFS of patients treated as per TuPro recommendations. **a**, Best response (CR, PR, SD, PD) in patients under palliative treatments (SOC and beyond SOC) in the Tumor Profiler cohort (TuPro, *n* = 82, 4 not evaluable)**. b**, Best response (CR, PR, SD, PD) in patients under palliative at least third line treatment in the TuPro cohort (*n* = 37). **c**, Median PFS in months in patients receiving palliative treatments (SOC and beyond SOC) in the TuPro cohort (*n* = 86). **d**, Median PFS in months in patients receiving palliative at least third line treatment in the TuPro cohort (*n* = 37).

The palliative SOC and beyond SOC TuPro participants (*n* = 86 lines of treatment) achieved a combined disease control rate (CR, PR or SD) of 61.0% (50 of 82, 4 not evaluable) (Fig. 5a). For the subgroups of patients who had already received at least three treatment lines, the disease control rate reached 56.8% (21 of 37) (Fig. 5b). Survival analysis showed a median progression-free survival (PFS) for patients in the palliative (both SOC and beyond SOC) of 6.04 months, (95% confidence interval (CI), 3.75–12.06) (Fig. 5c) and of 5.35 months (95% CI, 2.89–12.06) for subjects who were given at least three lines of treatment (*n* = 37) (Fig. 5d).

**Extended Data Fig. 4:**
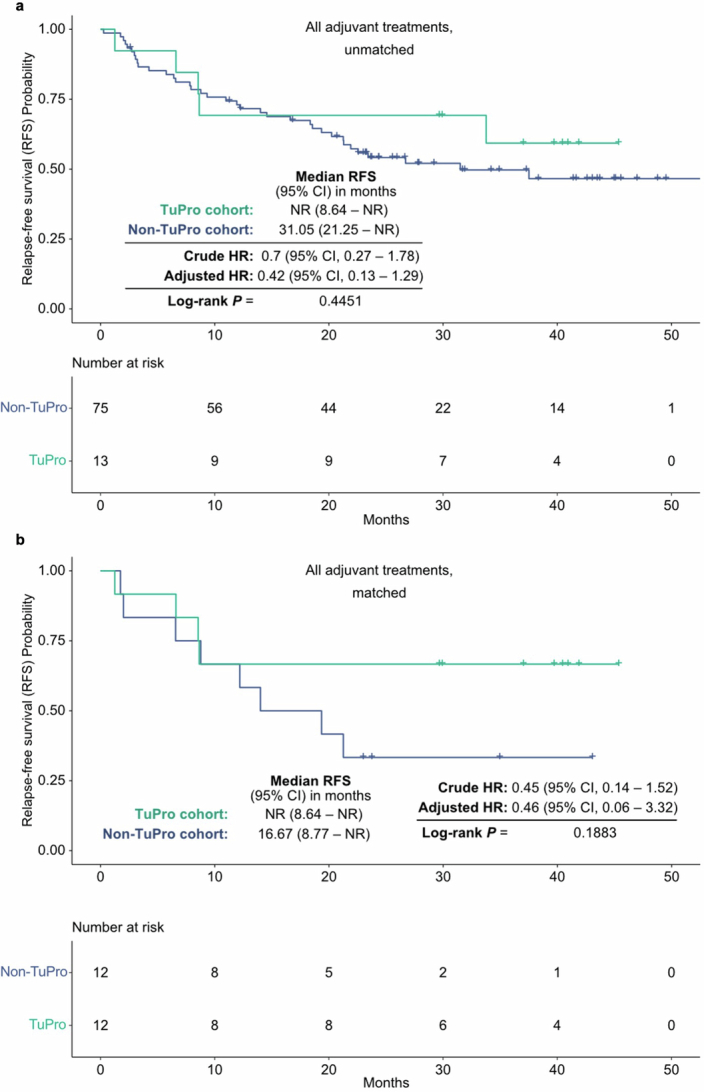
Adjusted relapse-free survival of adjuvant patients treated as per TuPro recommendation versus non-TuPro subjects. **a**, Kaplan-Meier curve and adjusted relapse-free survival (RFS) in months, crude hazard ratio, adjusted hazard ratio and Log-rank P-value for the comparison between unmatched adjuvant treatments. **b**, Kaplan-Meier curve and adjusted RFS in months, crude hazard ratio, adjusted hazard ratio and Log-rank P-value for the comparison between matched adjuvant treatments.

**Extended Data Fig. 5:**
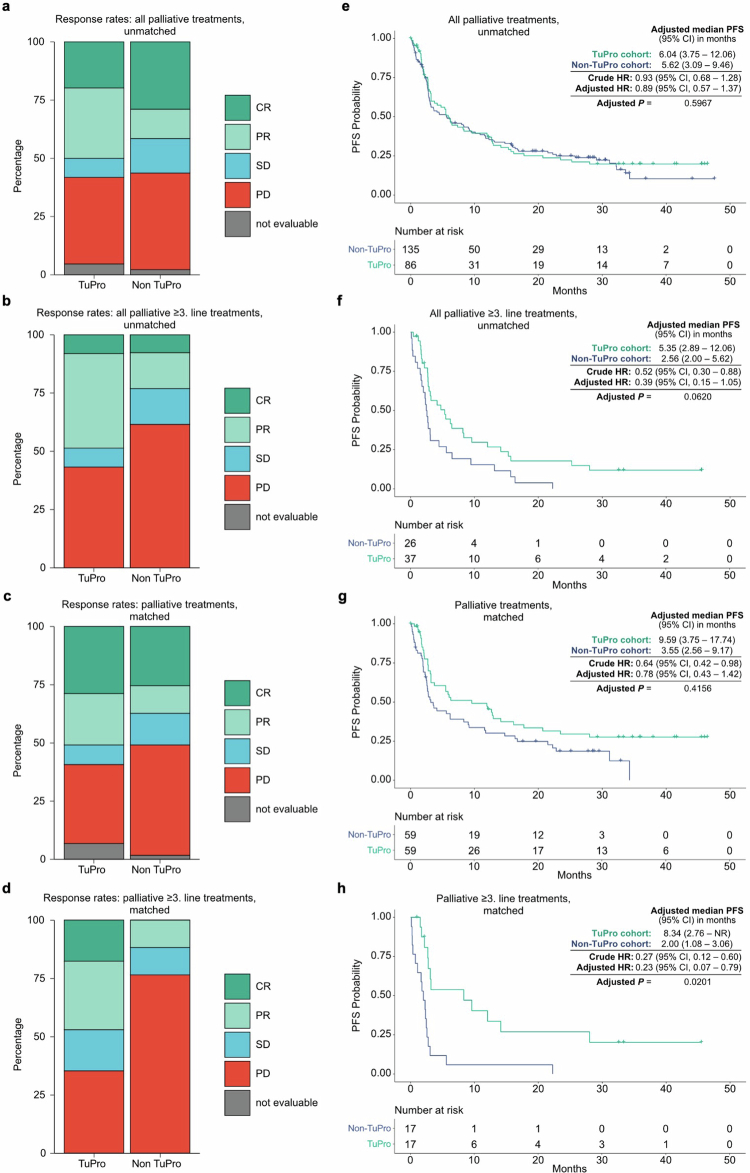
Response rates and adjusted PFS of patients treated in the palliative SOC or beyond SOC cohort as per TuPro recommendations versus non-TuPro subjects. **a**, Best response (complete response (CR), partial response (PR), stable disease (SD), progressive disease (PD)) in unselected, palliative treatments (SOC and beyond SOC) in the Tumor Profiler cohort (TuPro, *n* = 86) and the non-TuPro cohort (*n* = 132, 3 not evaluable). Combined disease control rate in the TuPro cohort of 61.0% (50/82, 4 not evaluable) vs. 57.6% (76/132, 3 not evaluable) in non-TuPro patients. Adjusted odds ratio of 1.54 (95% confidence interval (CI), 0.75 – 3.16). **b**, Best response (CR, PR, SD, PD) in unselected, palliative ≥ 3rd treatment line in the TuPro cohort (*n* = 37) and the non-TuPro cohort (*n* = 26). Combined disease control rate in the TuPro cohort of 56.8% (21/37) vs. 38.5% (10/26) in non-TuPro patients. Adjusted odds ratio of 3.67 (95% CI, 0.79 – 17). **c**, Best response of matched TuPro (*n* = 59) and non-TuPro (*n* = 59) palliative treatments (SOC and beyond SOC). Combined disease control rate in the TuPro cohort of 63.6% (35/55, 4 not evaluable) compared to 51.7% (30/58, 1 not evaluable), respectively. Adjusted odds ratio of 1.74 (95% CI, 0.68 – 4.45). **d**, Best response of matched TuPro (*n* = 17) and non-TuPro (*n* = 17) palliative ≥ 3rd treatment line. Combined disease control rate in the TuPro cohort of 64.7% (11/17) vs. 23.5% (4/17), respectively. Adjusted odds ratio of 7.18 (95% CI, 0.60 – 85.61). **e, f**, Kaplan-Meier curves and adjusted Progression free survival (PFS) in months, crude hazard ratio, adjusted hazard ratio and Log-rank *P*-value for the comparison between unmatched treatments: **e**, palliative (SOC and beyond SOC) TuPro cohort (n = 86 treatment lines) and non-TuPro cohort (*n* = 135 treatment lines): 6.04 months, (95% CI, 3.75 – 12.06) TuPro cohort vs. 5.62 months (*n* = 135, 95% CI, 3.09 – 9.46) in the non-TuPro cohort (crude hazard ratio (HR) of 0.93 (95% CI, 0.68 – 1.28), *P* = 0.5967, adjusted HR 0.89 (95% CI, 0.57 – 1.37). **f**, patients receiving ≥ 3rd treatment line in the TuPro cohort (*n* = 37) and non-TuPro cohort (*n* = 26): 5.35 months (95% CI, 2.89 – 12.06) vs. 2.56 months (*n* = 26, 95% CI, 2.00 – 5.62) (crude HR 0.52 (95% CI, 0.30 – 0.88), *P* = .0602, adjusted HR 0.39 (95% CI, 0.15 – 1.05), respectively. **g, h**, Kaplan-Meier curves and adjusted PFS in months, crude hazard ratio, adjusted hazard ratio and Log-rank *P*-value for the comparison between matched treatments: **g**, matched palliative (SOC and beyond SOC) TuPro cohort (*n* = 59 treatment lines) and non-TuPro cohort (*n* = 59 treatment lines): 9.59 months (95% CI, 3.75 – 17.74) vs. 3.55 months (95% CI, 2.56 – 9.17), respectively (adjusted HR of 0.78 (95% CI, 0.43 – 1.42), *P* = .4156. **h**, matched patients receiving ≥ 3 treatment line in the TuPro cohort (*n* = 17) and non-TuPro cohort (*n* = 17): 8.34 months (95% CI, 2.76 – NR) vs. 2.0 months (95% CI, 1.08 – 3.06), respectively (adjusted HR of 0.23 (95% CI, 0.07 – 0.79), *P* = .0201. CI, 95% confidence interval; HR, hazard ratio.

To further evaluate clinical benefit of the TuPro diagnostic pipeline, we retrospectively, in an exploratory way, compared outcomes of patients in the TuPro melanoma cohort with patients with melanoma from the same centers and period who were not part of the TuPro project (non-TuPro patients) (Extended Data Figs. 4 and 5, Extended Data Table 1 and Extended Data Table 2). To minimize potential bias, we performed a combined exact (treatment line, intention of treatment, clinical stage, brain metastases status) and propensity score matching (Methods), which resulted in comparable subgroups of 12 adjuvant treatments, 59 palliative treatments and 17 treatments with at least three treatment lines for TuPro and non-TuPro patients each (Supplementary Figure M1 and Extended Data Table 2).

The matched analysis of adjuvant patients (*n* = 12 matched treatments) showed no significant differences in relapse-free survival (RFS) (adjusted hazard ratio (HR) 0.46 (95% CI, 0.06–3.32) (Extended Data Fig. 4 and Extended Data Table 2). For the matched palliative groups (*n* = 59 matched treatments), median PFS in TuPro subjects reached 9.59 months (95% CI, 3.75–17.74) and 3.55 months (95% CI, 2.56–9.17) for non-TuPro subjects (adjusted HR of 0.78 (95% CI, 0.43–1.42), adjusted *P* = 0.4156) (Extended Data Fig. 5g). Within these matched groups, the disease control rate was 63.6% (35 of 55, 4 not evaluable) in the TuPro cohort compared to 51.7% (30 of 58, 1 not evaluable) in the non-TuPro cohort (Extended Data Fig. 5c). For subjects who had received at least three treatment lines (*n* = 17 matched treatments) the median PFS in the TuPro cohort was 8.34 months (95% CI, 2.76–NR) compared to 2.0 months (95% CI, 1.08–3.06) in the non-TuPro cohort (adjusted HR of 0.23 (95% CI, 0.07–0.79), adjusted *P* = 0.0201) and a disease control rate of 64.7% (11 of 17) compared to 23.5% (4 of 17) (Extended Data Fig. 5d,h and Extended Data Table 2). Overall, our exploratory analysis suggests an added value for heavily pretreated patients.

## Discussion

The TuPro project is a pioneering, prospective, multicohort precision oncology project, integrating cutting-edge technologies for the detailed and comprehensive investigation of tumor biopsies at an unprecedented level^18^. Here we report several clinical-translational endpoints of the project. First, we successfully generated high-quality molecular datasets from melanoma tumor biopsies using two standard (DigiPath and NGS) and seven experimental, mostly single-cell, high-throughput technologies (Pharmacoscopy, 4iDRP, IMC, CyTOF, proteotyping, scRNA-seq and scDNA-seq), which offer distinct and complementary perspectives on tumor biology. Second, we achieved a clinically meaningful turnaround time from sample acquisition to analysis and recommendation to the MTB of 4 weeks. This fast diagnostic loop supports the notion that incorporation of multiomics technologies into clinical oncology is feasible. Third, we successfully established a data-to-treatment pipeline that presented actionable insights gained from multimodal analysis to the MTB, facilitating actual clinical decision-making. Notably, the integration of MTBs into clinical decision-making correlates with improvements in various outcome measures^28^. Nevertheless, randomized controlled trials that formally assess these results need to be conducted in the future.

The treatment landscape for advanced melanoma has changed considerably over the past decade. The introduction of ICIs targeting PD1, CTLA-4 and LAG-3, as well as therapies addressing dysregulated and oncogenic MAPK pathway alterations (for example, *BRAF* and *MEK* mutations), has led to an unprecedented improvement in overall survival. Landmark clinical trials have demonstrated in first line striking ORRs of up to 58% and 68% for ICIs^29,30^ and BRAF and MEK inhibitors^31^, respectively. The 60% ORR observed in the TuPro palliative SOC group of first and further line patients compares favorably with published data. Notably, *BRAF* class I mutations currently are the only predictive biomarker used to determine eligibility for targeted therapy options^1,2^. However, single biomarker strategies have limitations, which are reflected by (1) a considerable number of patients with poor responses; (2) several negative high-profile clinical trials employing this strategy^7,32,33^; (3) genotype-matched trials (for example, MatchMel, SHIVA, MOSCATO, IMPACT/COMPACT) resulting in low target-drug matching rates (5–30%) or limited availability of interventional trials, and overall poor outcomes (ORR, 11–19%)^34–39^, and (4) single genetic alterations capturing only a fraction of the vastly complex biology of tumors. Both the successes and limitations outlined above form a strong rational for an expanded biomarker and/or assay panel to enable an in-depth mechanism-based therapy prediction for individual patients.

The TuPro project integrated various layers of molecular and cellular information, encompassing phosphorylation, cell–cell interactions, characteristics of the tumor microenvironment, inflammatory/immune markers, and ex vivo drug response. These factors are anticipated to play an important role in advanced and personalized predictive decision-making processes^40–42^. In the beyond SOC group, individual therapy recommendations were generated based on a larger number of individual markers compared to the palliative SOC group (average five versus four, respectively). For both groups of patients, this multimarker approach resulted in clinical meaningful responses in 38% of patients beyond SOC compared to 60% in the palliative SOC group. Although markers used to inform clinical decision-making within the TuPro project are biased toward known mechanisms and available drugs, the swift adoption of the expanded marker panel shows that clinicians rapidly acquire confidence in new readouts to support therapy. The project incorporated drugs that are not traditionally used in clinical care for melanoma patients. Therefore, the response rates in the beyond SOC TuPro cohort compare favorably when viewed from the perspective of other tumor-agnostic or phase I/II clinical trials, where substantial variations in ORR rates of 2–53% and median PFS of 5–7 months have been observed^43–46^.

Our analysis comparing the TuPro cohort with a synchronous non-TuPro melanoma cohort suggests that omics-guided treatments, such as in the TuPro project, result in comparable or better outcomes in terms of disease control rates and PFS, thus providing a rationale to further explore the analysis of -omics data as a means of therapy prediction in patients with melanoma. These data advocate that the expanded marker assessment via multiomics and functional technologies may contribute to address an unmet clinical need for difficult-to-treat patient populations. The ever-expanding range of therapeutic options necessitates a comprehensive understanding of tumor biology and individual patient characteristics. The TuPro project is uniquely positioned to achieve both immediate patient benefits and biological discoveries at the cohort level, which are currently under investigation.

We recognize several limitations of the analyses described and opportunities to improve future study designs. The current approach of selecting markers for treatment decisions by human experts cannot capture all potentially relevant information. The poor response in several patients treated with individualized therapies may reflect this shortcoming. The advent and rapid evolution of artificial intelligence and machine learning methods will probably need to be leveraged to translate the extensive complexity of TuPro datasets into enhanced clinical benefit. Further process optimization and efficacy improvements will facilitate the full implementation of all available technologies. Interpretations regarding clinical utility of serial biopsies are limited by heterogenous results and small sample size. The retrospective comparison of TuPro and non-TuPro patient limits extrapolation and generalizability. Although we attempted to provide comparable results using matched cohorts, the project was first and foremost a feasibility study. One main goal was to identify patients with melanoma most likely to benefit from extended TuPro analysis. To this end, patient inclusion was broad, leading to a heterogenous population. Benefit was seen mostly in the beyond SOC cohort in patients failing several lines of treatment. Given key uncertainties (patients who are most likely to benefit, expected effect size, technology performance, feasibility of clinical translation of findings by the MTB) a randomized trial would not have been feasible at the time the project was designed. Clearly blinded, randomized trials are needed to confirm our findings in a next step.

Based on our calculations, the per patient cost of analysis in the beyond SOC setting is above the current coverage limits by health insurance for a NGS panel (up to 4,000 Swiss francs in Switzerland) yet only exceeds this limit by a factor of 1.8 when using a limited number of technologies that would possibly deliver the same answers. In addition, we demonstrate that at costs comparable to those covered by insurance (4602 Swiss francs), we were able to successfully profile 38 out of 39 patients (97%) within the SOC setting. The current cost estimates fall within a range that allows for evaluation of potential benefits through a randomized trial. In conclusion, TuPro has successfully pioneered the integration of predictive polymarker assessment with marker-driven, highly individualized treatments, demonstrating the feasibility of complex and comprehensive data-driven approaches to improve patient care.

## Methods

### Patient selection, data acquisition and sample processing

TuPro was conducted as a multicentric precision oncology project according to the Swiss Act and Ordinance on Human Research (HFG and HFV) with approval of, and in compliance with, regulatory authorities (ethics committees of northwestern Switzerland, EKNZ, and of Zurich, KEK). Adult patients diagnosed with any subtype of melanoma, both treatment-naive and those treated either in the adjuvant or palliative setting were eligible for inclusion. The project was conducted at the Department of Dermatology, University Hospital Zurich, at the Department of Oncology, University Hospital Basel and at the Department of Oncology and Hematology, Kantonsspital Baselland, Switzerland, from January 2019 until November 2020 (EC-ID, 2018-02050, 2021-01584) in accordance with the applicable legal and institutional standards. The project was registered with clinicaltrials.gov (NCT06463509). For each specific (single agent or combined) off-label treatment, regardless of whether the Tumor Board decision was based on routine patient data or the MSR, the project allowed inclusion of up to five patients. If the same off-label treatment was proposed to a sixth patient, the patient had to be referred to an interventional trial. Due to the diversity of tumor board recommendations, the number of more than five identical off-label treatments was not reached in the cohort we report here. Treatments were not given as part of the study. Separate consents were required for any selected treatment regimens. All decisions regarding treatment regimens were made by the treating physician and, although it could be influenced by the TuPro data, the final treatment selection for each patient was at the sole discretion of the treating physician based on their experience and expertise. The demographical, clinical and pathological features of eligible patients, including age range, sex, melanoma subtype, stage (according to the American Joint Committee on Cancer, eighth edition), together with previous treatments and treatments after biopsy collection, were obtained from our institutional database (Klinikinformationssystem (KISIM)) and entered in the good-clinical-practice-compliant database secuTrial v.6.1.2.5, 2021 (Extended Data Table 1). Sex- and gender-based analyses were not conducted due to the small sample size and the feasibility-focused nature of the study, which did not include sex- or gender-specific stratification. Eligible female and male patients (*n* = 116), age range 20–89 years, underwent tissue biopsies as part of their diagnostic workup. Leftover tissue samples from routine diagnostics were used in this study, with participants providing project-specific written informed consent. Participants did not receive compensation for their involvement in the study. Experienced pathologists evaluated the biopsy samples for viability and tumor cell content (requiring a minimum of 20% viable tumor cells). Samples meeting these quality criteria were further processed in a central laboratory and underwent paraffin embedding or single-cell dissociation. On average, 2,000,000 viable single tumor cells per sample were recovered. Samples were transferred to the following analytical platforms (referred to as technology nodes) for further processing and analysis: CyTOF^22^, IMC^21^, scRNA-seq^20^, scDNA-seq^19^, mass spectrometry proteotyping^23–25^, Pharmacoscopy^11,26^ or 4iDRP^27^. To establish technical feasibility, robustness, and reproducibility of sample and data handling logistics, ten (*n* = 10) samples were initially analyzed and results were not discussed at the MTB. The remaining samples (*n* = 116) were analyzed within the TuPro framework and were reported to the MTB via an MSR.

As a feasibility project, outcomes were broadly defined as follows:

1. Feasibility outcomes: types of molecular information and combinations of molecular information from the biotechnology domain that the pre-TB considers as useful for making a treatment recommendation beyond routine diagnostics (including routine pathology and panel NGS testing).
2. Classification of proposed treatment options based on TuPro measurements: (a) on-label treatment with molecular matched treatment (label of the Swiss Agency for Therapeutic Products, SwissMedic, as a reference) ± radiotherapy or chemotherapy; (b) treatment with classical chemotherapy ± radiotherapy (on label if label available); (c) referral to a suitable clinical trial; (d) off-label treatment (SwissMedic label as a reference) with molecular matched treatment or immunotherapy ± radiotherapy or chemotherapy; (e) off-label treatment (authorization in countries with comparable approval and control systems for medicinal products as defined by SwissMedic) with molecular matched treatment or immunotherapy ± radiotherapy or chemotherapy; (f) immunotherapy; (g) no active anti-tumor treatment (BSC).
3. Clinical outcomes: (a) best response to treatment, assessed radiologically after treatment initiation, classified according to RECIST criteria; (b) PFS as the duration in months between the date of treatment initiation (first medication intake) and the date of the first radiologically confirmed progression, if progression occurred.

The TuPro protocol is available upon request to the Tumor Profiler Center (TPC) (nicola.miglino@usz.ch). To comply with applicable laws and regulations (the Swiss Human Research Act), all deidentified clinical data relevant to this publication are provided as supporting information to the paper. Access to the patient-level clinical and biological data presented at the MTBs will be granted to registered users listed on the data access agreement with the TPC within 4 weeks of receipt of the Data Access Agreement, provided that the applicant submits all necessary ethics committee approvals and supporting documents needed to meet the requirements of the agreement. Data access can be requested by contacting the TPC (nicola.miglino@usz.ch). The user institution agrees to destroy or discard the data once it is no longer used for the project, and in cases where data must be archived, they must be deleted within 10 years of the project’s completion. If data have not been archived, they must be deleted no later than 2 years following the completion of the project. An extension to this period can be provided upon request to the TPC leadership. Data sharing is subject to honoring patient privacy and data integrity.

### Data analysis and generation of MSR

The readouts obtained from the clinical standard technologies and the TuPro experimental technologies generated up to 500 Gb of data for each tumor sample. The goal was to analyze and summarize this vast amount of data in a clinically meaningful manner. We aimed to identify datapoints that were valuable for decision-making and determine the necessary steps to ensure the usability and reliability of therapy predictions to enhance patient outcomes. To this end, detailed discussions between members from various technology nodes, data scientists, molecular pathologists and clinicians in the pre-TB condensed this information into the MSR. The report summarized all drugs recommended by NGS, Pharmacoscopy or 4iDRP, plus the supporting or counter-indicating evidence from the other technologies (CyTOF, IMC, scRNA-seq, scDNA-seq, proteotyping, DigiPath). Recommendations were based on associations between drugs and markers, including drug–gene, drug–signaling and drug–immune environment pairs. These associations were supported by varying levels of evidence (for example, OncoKB, clinical guidelines, preclinical evidence). For instance, response to MEK inhibitors (for example, trametinib) was associated with mutated *NRAS*, *BRAF*, *GNAQ/GNA11*, *NF1* and/or an activated MAPK pathway, indicated by pERK. ICIs (for example, nivolumab) response was linked to HLA expression, an inflammatory tumor phenotype and checkpoint expression. The TuPro protocol did not stipulate how the MSR should be used by the MTB. However, clinicians adhered to available and applicable guidelines such as from the European Society for Medical Oncology (ESMO) and the National Comprehensive Cancer Network (NCCN), which included recommendations on off-label medication use and the regulatory standards for patient allocation to clinical projects or trials.

### Diagnostic evaluation levels

To assess the additional value of TuPro data in conjunction with standard technologies such as NGS and digital pathology, a stepwise evaluation process at the pre-TB was established. Level 1 included detailed clinical information, patient history, genetic tumor data (*BRAF*, *NRAS*, *c-KIT*) and insights from DigiPath. For level 2, the results of a large NGS panel of 324 genes (FoundationOne CDx) were incorporated into the assessment of level 1. Level 2 was considered the most comprehensive SOC evaluation at the time for patients with advanced melanoma. Level 3 additionally implemented data provided by the seven experimental TuPro technology nodes (CyTOF, IMC, scRNA-seq, scDNA-seq, proteotyping, Pharmacoscopy, 4iDRP). Findings for level 3 were summarized by the TuPro reporting and clinical teams within the framework of the pre-TB. Only medications approved by SwissMedic (on or off label, around 180 at the time of the project) or drugs under investigation in clinical trials at one of the TuPro centers (*n* = 20) were considered. The complete MSR for each participant was communicated to the MTB to inform treatment recommendations.

### Assessment of clinical usefulness

The assessment of clinical usefulness for each marker and technology was carried out in the TuPro application cohort (*n* = 93 patients and 99 lines of therapy) (Fig. 1). This population consists of patients who were discussed in both pre-TB and MTB, had all levels of treatment recommendations recorded, received anti-cancer treatment and had available outcome assessments. The TuPro application cohort was categorized into three groups: (1) adjuvant therapy (decision between two standards, TKI therapy for *BRAF*-mutant tumors versus immunotherapy, and one trial-associated therapy, anti-CTLA-4 + anti-PD1), (2) palliative therapy within the SOC (that is, immunotherapy, targeted therapy, talimogene laherparepvec, chemotherapy) and (3) palliative therapy beyond the SOC (interventional trials, off-label use, or off-label reintroduction of approved therapies after confirmed progression upon treatment with these agents). Two patients were sequentially included first in the SOC and later in the beyond SOC group. Of note, in this diagnostic proof-of-concept project, patients were not preselected to allow for TuPro-directed treatment, that is, there was no inclusion criterion regarding life expectancy.

Treatment decisions were considered TuPro-driven if ≥50% of the drugs administered were selected based on markers reported by a TuPro technology node^15^. This also applied to patients who were already undergoing treatment, when alterations reported by TuPro technologies confirmed ≥50% of the drugs in the ongoing treatment regimen.

### Cost analysis

The costs for multiomics profiling were assessed as marginal costs of production, that is, the cost of one additional analysis on an otherwise fully funded analysis platform running at full capacity. The market price was used for the cost of the NGS genomic marker test. For experimental technologies, costs were assessed using Swiss research prices (Swiss Personalized Oncology program costs, DigiPath) accounting for personnel costs (analysis time and full-time-equivalent salary costs) as well as costs of reagents.

### Safety and outcome assessment

All patients receiving therapy in the TuPro project underwent regular assessments following standard operating procedures at the participating institutions. Response assessment was conducted every 2–3 months using medical imaging scored via RECIST v.1.1 criteria. Full patient follow-up extended for the duration of the project and an additional 6 months after the project’s conclusion. PFS was calculated from the date of therapy initiation to documented disease progression or until the last follow-up for nonprogressed patients with a cutoff date on 31 May 2023. Clinical endpoints were defined in the project protocol and included the number of cases in which the MTB deemed the MSR useful, number of cases in which treating physicians found the MTB recommendations beneficial, specific information (markers) considered relevant by the MTB for treatment recommendations beyond routine diagnostics (DigiPath, NGS), classification of proposed treatment options (in-label, off-label, trial, BSC or no therapy), ORR (which included CR and PR), disease control rate (CR, PR, SD), duration of response, time to next therapy or BSC and the proportion of patients who discontinued treatment due to toxicity.

### Comparison cohort and matching

In an exploratory, retrospective analysis, outcomes in the TuPro cohort were compared to those of melanoma patients who were not recruited into TuPro but received diagnostic assessment and subsequent systemic therapy at the TuPro centers over a comparable period (1 February 2019 to 1 July 2021) (Extended Data Figs. 4 and 5, Extended Data Table 2 and Supplementary Table M4). The comparison cohort included 141 patients followed until 31 May 2023, who received 216 therapies (6 neoadjuvant, 75 adjuvant, 96 palliative SOC, 39 beyond SOC). In addition to comparing unmatched RFS, PFS and response rates, a matched analysis was performed to establish comparable subgroups and reduce bias.

The TuPro treatments were matched (1:1) to the non-TuPro treatments using a genetic matching algorithm (MatchIt package^47^) without replacement with propensity scores estimated with a multivariate logistic regression model including the following pretreatment variables: Charlson Comorbidity index, Eastern Cooperative Oncology Group score (0–5), histologic subtype, lactate dehydrogenase levels at baseline, tumor mutational burden and pathogenic mutational status of *BRAF* (yes/no). Treatments with missing matching variables were excluded before the matching (*n* = 14 non-TuPro treatments). To optimize cohort comparability, we combined propensity score matching with exact matching for all palliative comparisons with the following variables: clinical stage (I–IV), intention of treatment (SOC, beyond SOC), treatment line (1–8) and presence of brain metastases (yes/no). In the adjuvant group, exact matching was performed based solely on clinical stage, as treatment intention, treatment line and brain metastases are not applicable in this setting. This led to 59 palliative treatments matched (leaving 27 TuPro and 76 non-TuPro treatments unmatched), 17 palliative at least three treatment lines matched (leaving 20 TuPro and 9 non-TuPro treatments unmatched) and 12 adjuvant treatments matched (leaving 1 TuPro and 63 non-TuPro treatments unmatched).

### Statistical analysis

Unadjusted rates and adjusted odds ratios were calculated for the binary outcome of either disease control (CR, PR, SD) or progression (PD). Mixed-effects adjusted logistic regression models were used to explore the association between disease control and TuPro participation. The models for the unmatched analysis included the following variables as covariates: Eastern Cooperative Oncology Group (binary variable, ≥2), lactate dehydrogenase at baseline, presence of brain metastases (yes/no), age, clinical stage (III or IV), intention of treatment (adjuvant, SOC, beyond SOC) and treatment line (< or ≥ third line). In the subanalysis of treatments for subjects treated with three or more lines of therapy, the treatment line and clinical stage was excluded as a covariate. In the models for the matched cohorts, the propensity score served as a covariate. Median RFS and PFS were estimated using a Cox model with frailty to account for patients receiving several treatments. A mixed-effects Cox proportional-hazards regression model was used to calculate adjusted HRs and *P* values comparing RFS and PFS between TuPro versus non-TuPro treatments with random effects accounting for patients with several included treatments. The unmatched analysis utilized the same covariates as the corresponding best treatment response models, and the matched analysis included the propensity score as a covariate. In the unmatched adjuvant analysis, presence of brain metastases, intention of treatment and treatment line were excluded as covariates. The proportional hazard assumption was tested with the Schoenfeld residual test, for which variables with a *P* value > 0.05, were considered to fulfill the assumption. All analyses tested the null hypothesis using a two-sided 0.05 significance level and included 95% CI calculations. R (R Core Team, v.4.4.1) was used for all statistical analyses.

### Reporting summary

Further information on research design is available in the Nature Portfolio Reporting Summary linked to this article.

## Online content

Any methods, additional references, Nature Portfolio reporting summaries, source data, extended data, supplementary information, acknowledgements, peer review information; details of author contributions and competing interests; and statements of data and code availability are available at 10.1038/s41591-025-03715-6.

## Supplementary information

Supplementary Information

Reporting Summary

Supplementary Data Deidentified clinical data table.

Supplementary Table Dictionary to the clinical table.

## Figures and Tables

**Extended Data Table 1:**
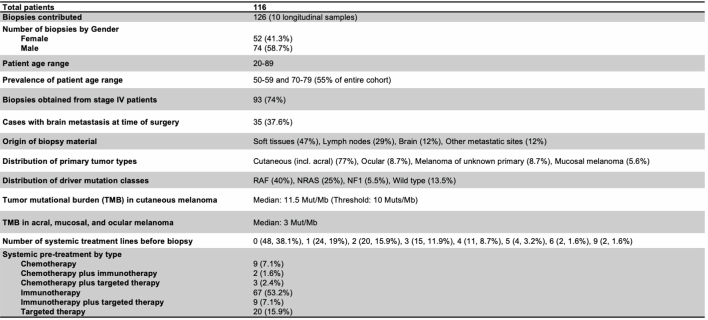
TuPro melanoma cohort (n = 116) patient and sample characteristic

**Extended Data Table 2:**
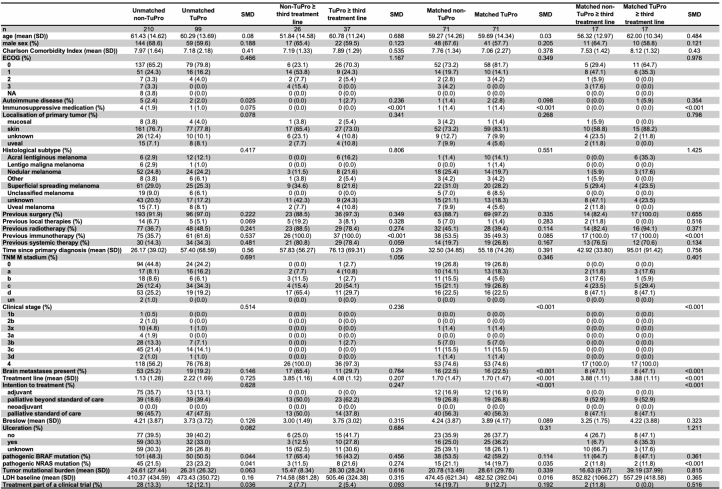
Patient characteristics of TuPro and Non-TuPro treatments for unmatched and matched cohorts and ≥ third treatment line subgroups. SD: standard deviation, SMD: standardized mean difference

## Data Availability

To comply with applicable laws and regulations (the Swiss Human Research Act), all deidentified clinical data relevant to this publication are provided as supporting information to the paper. Access to the patient-level clinical and biological data presented at the MTBs will be granted to registered users listed on the data access agreement with the TPC within 4 weeks of receipt of the Data Access Agreement, provided that the applicant submits all necessary ethics committee approval and supporting documents needed to meet the requirements of the agreement. Data access can be requested by contacting the TPC (nicola.miglino@usz.ch). The user institution agrees to destroy or discard the data once it is no longer used for the project, and in cases where data must be archived, it must be deleted within 10 years of the project’s completion. If data has not been archived, it must be deleted no later than 2 years following the completion of the project. An extension to this period can be provided upon request to the TPC leadership. Data sharing is subject to honoring patient privacy and data integrity.
