# Publisher Correction: Feasibility of multiomics tumor profiling for guiding treatment of melanoma

**DOI:** 10.1038/s41591-025-03904-3

**Published:** 2025-08-01

**Authors:** Nicola Miglino, Nora C. Toussaint, Alexander Ring, Ximena Bonilla, Marina Tusup, Benedict Gosztonyi, Tarun Mehra, Gabriele Gut, Francis Jacob, Stephane Chevrier, Kjong-Van Lehmann, Ruben Casanova, Andrea Jacobs, Sujana Sivapatham, Laura Boos, Parisa Rahimzadeh, Manuel Schuerch, Bettina Sobottka, Natalia Chicherova, Shuqing Yu, Rebekka Wegmann, Julien Mena, Emanuela S. Milani, Sandra Goetze, Cinzia Esposito, Jacobo Sarabia del Castillo, Anja L. Frei, Marta Nowak, Anja Irmisch, Jack Kuipers, Monica-Andreea Baciu-Drăgan, Pedro F. Ferreira, Franziska Singer, Anne Bertolini, Michael Prummer, Ulrike Lischetti, Tumor Profiler Consortium, Nicola Miglino, Nora C. Toussaint, Ximena Bonilla, Marina Tusup, Gabriele Gut, Francis Jacob, Kjong-Van Lehmann, Ruben Casanova, Andrea Jacobs, Sujana Sivapatham, Manuel Schuerch, Bettina Sobottka, Natalia Chicherova, Shuqing Yu, Rebekka Wegmann, Julien Mena, Emanuela S. Milani, Sandra Goetze, Cinzia Esposito, Jacobo Sarabia del Castillo, Anja L. Frei, Marta Nowak, Anja Irmisch, Jack Kuipers, Pedro F. Ferreira, Franziska Singer, Anne Bertolini, Michael Prummer, Ulrike Lischetti, Melike Ak, Faisal S. Al-Quaddoomi, Silvana I. Albert, Jonas Albinus, Ilaria Alborelli, Sonali Andani, Per-Olof Attinger, Monica-Andreea Baciu-Drăgan, Daniel Baumhoer, Beatrice Beck-Schimmer, Lara Bernasconi, Lars Bosshard, Byron Calgua, Stéphane Chevrier, Ricardo Coelho, Maya D’Costa, Esther Danenberg, Natalie R. Davidson, Stefanie Engler, Martin Erkens, Katja Eschbach, André Fedier, Joanna Ficek-Pascual, Bruno Frey, Linda Grob, Detlef Günther, Pirmin Haeuptle, Viola Heinzelmann-Schwarz, Sylvia Herter, Rene Holtackers, Tamara Huesser, Alexander Immer, Tim M. Jaeger, Alva R. James, Philip M. Jermann, André Kahles, Abdullah Kahraman, Werner Kuebler, Christian P. Kunze, Christian Kurzeder, Mitchell Levesque, Flavio C. Lombardo, Sebastian Lugert, Philipp Markolin, Martin Mehnert, Julian M. Metzler, Simone Muenst, Riccardo Murri, Charlotte K. Y. Ng, Stefan Nicolet, Monica Nunez Lopéz, Patrick GA Pedrioli, Salvatore Piscuoglio, Laurie Prélot, Natalie Rimmer, Mathilde Ritter, Christian Rommel, María L. Rosano-González, Natascha Santacroce, Ramona Schlenker, Petra C. Schwalie, Severin Schwan, Tobias Schär, Gabriela Senti, Wenguang Shao, Vipin T. Sreedharan, Stefan Stark, Daniel J. Stekhoven, Tanmay Tanna, Tinu M. Thomas, Markus Tolnay, Vinko Tosevski, Mustafa A. Tuncel, Audrey Van Drogen, Marcus Vetter, Tatjana Vlajnic, Sandra Weber, Walter P. Weber, Fabian Wendt, Norbert Wey, Mattheus HE Wildschut, Johanna Ziegler, Marc Zimmermann, Martin Zoche, Gregor Zuend, Rudolf Aebersold, Marina Bacac, Gerd Maass, Holger Moch, Michael Weller, Alexandre P. A. Theocharides, Markus G. Manz, Niko Beerenwinkel, Christian Beisel, Lucas Pelkmans, Berend Snijder, Bernd Wollscheid, Bernd Bodenmiller, Viktor H. Koelzer, Gunnar Rätsch, Reinhard Dummer, Andreas Wicki, Rudolf Aebersold, Marina Bacac, Gerd Maass, Holger Moch, Michael Weller, Alexandre P. A. Theocharides, Markus G. Manz, Niko Beerenwinkel, Christian Beisel, Lucas Pelkmans, Berend Snijder, Bernd Wollscheid, Viola Heinzelmann, Bernd Bodenmiller, Mitchell P. Levesque, Viktor H. Koelzer, Gunnar Rätsch, Reinhard Dummer, Andreas Wicki

**Affiliations:** 1 Department of Medical Oncology and Hematology, University of Zurich and University Hospital, Zurich, Switzerland; 2 NEXUS Personalized Health Technologies, ETH Zurich, Zurich, Switzerland; 3 SIB Swiss Institute of Bioinformatics, Lausanne, Switzerland; 4 Swiss Data Science Center SDSC, Zurich, Switzerland; 5 Department of Computer Science, Institute of Machine Learning, ETH Zurich, Zurich, Switzerland; 6 Department of Dermatology, University Hospital Zurich, University of Zurich, Zurich, Switzerland; 7 Department of Biomedicine, University Hospital Basel and University of Basel, Basel, Switzerland; 8 Department of Quantitative Biomedicine, University of Zurich, Zurich, Switzerland; 9 Department of Biology, RWTH Aachen, Aachen, Germany; 10 Department of Pathology and Molecular Pathology, University of Zurich and University Hospital, Zurich, Switzerland; 11 Department of Biology, Institute of Molecular Systems Biology, ETH Zurich, Zurich, Switzerland; 12 Department of Health Sciences and Technology, ETH Zurich, Zurich, Switzerland; 13 ETH PHRT Swiss Multi-Omics Center (SMOC), ETH Zurich, Zurich, Switzerland; 14 Department of Molecular Life Sciences, University of Zurich, Zurich, Switzerland; 15 Roche Pharmaceutical Research and Early Development, Roche Innovation Center, Zurich, Switzerland; 16 Department of Biosystems Science and Engineering, ETH Zurich, Basel, Switzerland; 17 Roche Diagnostics GmbH, MWG, Penzberg, Germany; 18 Department of Neurology, University Hospital and University of Zurich, Zurich, Switzerland; 19 Institute of Medical Genetics and Pathology, University Hospital Basel, Basel, Switzerland; 20 Biomedical Informatics, University Hospital Zurich, Zurich, Switzerland; 21 AI Center at ETH Zurich, ETH Zurich, Zurich, Switzerland; 22 Department of Biology, ETH Zurich, Zurich, Switzerland; 23 F. Hoffmann-La Roche Ltd, Basel, Switzerland; 24 University of Zurich, VP Medicine, Zurich, Switzerland; 25 University Hospital Zurich, Clinical Trials Center, Zurich, Switzerland; 26 University Hospital Basel, Basel, Switzerland; 27 Roche Pharmaceutical Research and Early Development, Roche Innovation Center, Basel, Switzerland; 28 Life Science Zurich Graduate School, Biomedicine PhD Program, Zurich, Switzerland; 29 ETH Zurich, Department of Chemistry and Applied Biosciences, Zurich, Switzerland; 30 Cantonal Hospital Baselland, Medical University Clinic, Liestal, Switzerland; 31 Max Planck ETH Center for Learning Systems, Zurich, Switzerland; 32 FHNW, School of Life Sciences, Institute of Chemistry and Bioanalytics, Muttenz, Switzerland; 33 University Hospital Basel, Department of Information- and Communication Technology, Basel, Switzerland; 34 University Hospital Basel, Brustzentrum, Basel, Switzerland; 35 University Hospital Zurich, Department of Gynecology, Zurich, Switzerland; 36 University of Bern, Department of BioMedical Research, Bern, Switzerland; 37 Roche Pharmaceutical Research and Early Development, Roche Innovation Center Munich, Roche Diagnostics GmbH, Penzberg, Germany; 38 University Hospital Basel, Brustzentrum and Tumorzentrum, Basel, Switzerland; 39 Department of Surgery, Brustzentrum, University Hospital Basel and University of Basel, Basel, Switzerland; 40 University Hospital Zurich, Zurich, Switzerland

**Keywords:** Melanoma, Cancer genomics, Tumour immunology, Tumour biomarkers, Cancer therapy

Correction to: *Nature Medicine* 10.1038/s41591-025-03715-6, published online 27 May 2025.

In the version of the article initially published, the far-right column of the lower panel in Fig. 2c was missing and has now been added, as seen in Fig. 1, below. Additionally, the sentence “L.P. is a founder and shareholder of Apricot Therapeutics - a precision oncology spin-off from the University of Zurich - based on 4i drug response profiling” was missing from the Competing interests statement and has now been added. These corrections have been made to the HTML and PDF versions of the article.

**Fig. 1:**
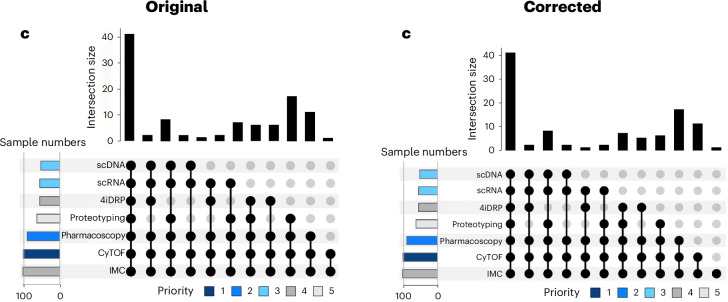
Original and corrected Fig. 2c.

