# Supplementary material for: Feasibility of multiomics tumor profiling for guiding treatment of melanoma
Source: Nat Med. 2025 May 27;31(7):2430–41. doi: 10.1038/s41591-025-03715-6 (PMC12283375; doi:10.1038/s41591-025-03715-6)
Supplement: Supplementary file 1 — Supplementary Information [file 41591_2025_3715_MOESM1_ESM.pdf]

nature medicine

Supplementary information https://doi.org/10.1038/s41591-025-03715-6

Feasibility of multiomics tumor profiling for guiding treatment of melanoma

In the format provided by the authors and unedited

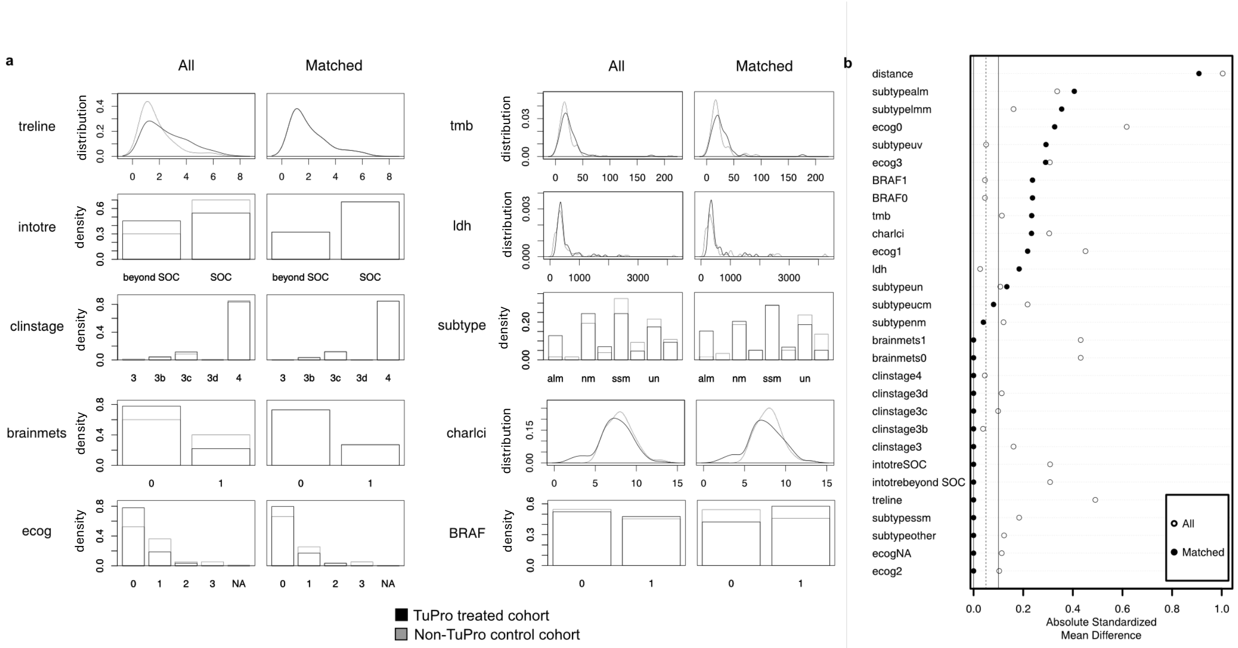

Supplementary Figure M1a: Matching covariates in the palliative setting. Density plots showing matching covariates for unmatched (All) palliative TuPro (*n*=86) and non-TuPro (*n*=130) cohorts and matched (Matched) palliative TuPro (*n*=59) and non-TuPro (*n*=59) cohorts. b, Love plots showing the standardized mean difference of matching covariates before (All) and after matching (Matched) of palliative TuPro and non-TuPro patients. treline, treatment line; intotre, intention of treatment; SOC, standard of care; clinstage, clinical stage; brainmets, presence of brain metastases; ecog, Eastern Cooperative Oncology Group performance status; tmb, Tumor mutational burden; LDH, Lactate dehydrogenase; subtype, histologic subtype; alm, acral lentiginous melanoma; nm, nodular melanoma; ssm, superficial spreading melanoma; un, unknown; lmm, lentigo malignant melanoma; ucm, unclassified melanoma; uv, uveal melanoma; other, other melanoma subtypes. The y-axis displays the density of observations for categorical variables and a smoothed distribution for continuous variables. LDH values are presented in units of U/L, while TMB is expressed as mutations per megabase.

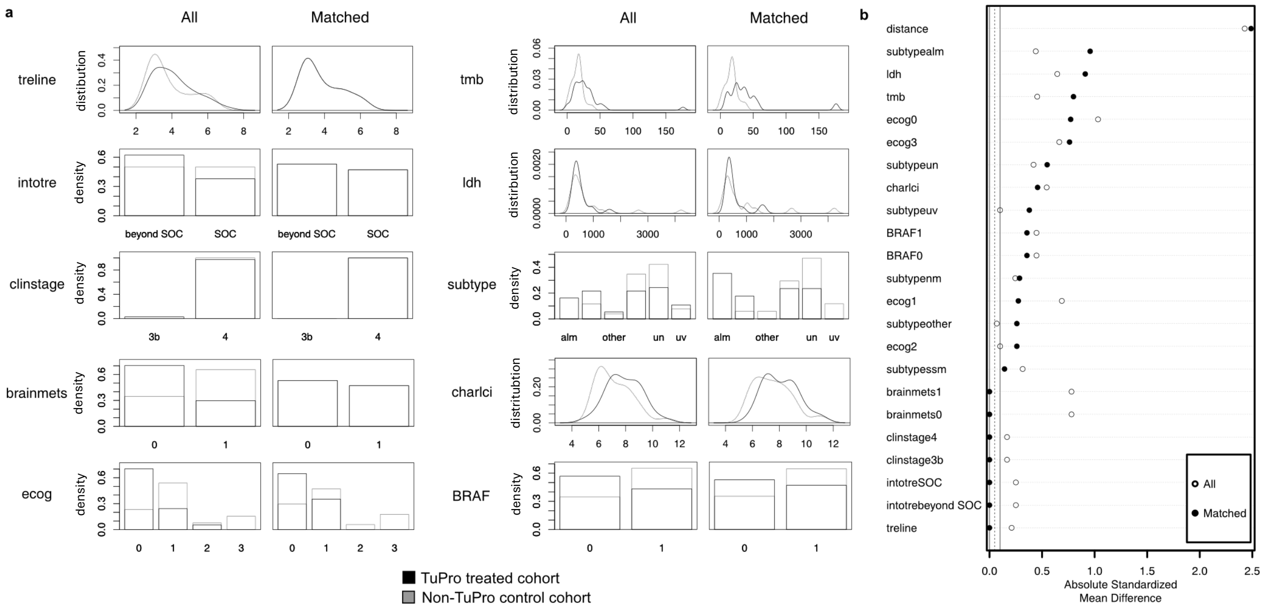

Supplementary Figure M1b: Matching covariates in the palliative ≥ 3rd treatment line setting. Density plots showing matching covariates for unmatched (All) palliative ≥ 3rd treatment line TuPro (*n*=37) and non-TuPro (*n*=26) cohorts and matched (Matched) palliative ≥ 3rd treatment line TuPro (*n*=17) and non-TuPro (*n*=17) cohorts. b, Love plots showing the standardized mean difference of matching covariates before (All) and after matching (Matched) of palliative ≥ 3rd treatment line TuPro and non-TuPro patients. treline, treatment line; intotre, intention of treatment; SOC, standard of care; clinstage, clinical stage; brainmets, presence of brain metastases; ecog, Eastern Cooperative Oncology Group performance status; tmb, Tumor mutational burden; LDH, Lactate dehydrogenase; subtype, histologic subtype; alm, acral lentiginous melanoma; nm, nodular melanoma; ssm, superficial spreading melanoma; un, unknown; lmm, lentigo malignant melanoma; ucm, unclassified melanoma; uv, uveal melanoma; other, other melanoma subtypes. The y-axis displays the density of observations for categorical variables and a smoothed distribution for continuous variables. LDH values are presented in units of U/L, while TMB is expressed as mutations per megabase.

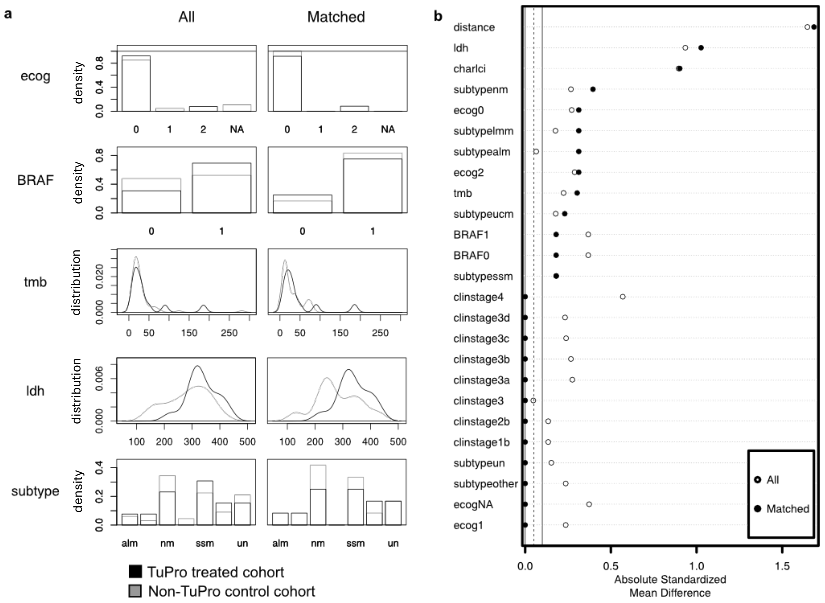

Supplementary Figure M1c: Matching covariates in the adjuvant setting. Density plots showing matching covariates for unmatched (All) adjuvant TuPro (n=13) and non-TuPro (n=67) cohorts and matched (Matched) adjuvant TuPro (*n*=12) and non-TuPro (*n*=12) cohorts. b, Love plots showing the standardized mean difference of matching covariates before (All) and after matching (Matched) of adjuvant TuPro and non-TuPro patients. treline, treatment line; intotre, intention of treatment; SOC, standard of care; clinstage, clinical stage; brainmets, presence of brain metastases; ecog, Eastern Cooperative Oncology Group performance status; tmb, Tumor mutational burden; LDH, Lactate dehydrogenase; subtype, histologic subtype; alm, acral lentiginous melanoma; nm, nodular melanoma; ssm, superficial spreading melanoma; un, unknown; lmm, lentigo malignant melanoma; ucm, unclassified melanoma; uv, uveal melanoma; other, other melanoma subtypes. The y-axis displays the density of observations for categorical variables and a smoothed distribution for continuous variables. LDH values are presented in units of U/L, while TMB is expressed as mutations per megabase.

Targeted technologies

Technology	Priority	Number of markers
NGS	T1	324
Digitial pathology	T2	9
CyTOF	C1	90
Pharmacoscopy	C2	60
4iDRP	C4	12
IMC	T4	49

Untargeted technologies

Technology	Priority	Number of markers
Proteotyping	T3, C5	~7,000
scRNA	C3	~16,000
scDNA	C3	~20,000

Supplementary Table M1: Data points (n>40,000) measured per technology node (n=9), considering every measurement. Data presentation is divided by targeted technologies (i.e., an a priori defined set of makers are measured) and untargeted technologies (i.e., whole transcriptome, genome and proteome analysis). T1-4, tissue priority, with 1 being the highest priority; C1-5, single cell suspension priority, with 1 being the highest priority.

Supplementary Table M2a: Marker equivalencies table for the TuPro adjuvant cohort

Marker	FMI	DigiPath	scRNA	scDNA	CyTOF	IMC	Proteotype	Pharmacoscopy	4iDRP
BRAF	1								
RAF1	1		1	1			1		
HLA-ABC			1		1	1	1		
IFN_gamma			1			1			
IFN_gamma+T_cells			1			1			
PD-1		1	1		1	1			
PD-L1		1	1		1	1			
TMB	1								
pERK					1	1			1
MEKi+BRAFi								1	1
T_cell_infiltration		1				1			
TLS		1				1			

Supplementary Table M2b: Marker equivalencies table for the TuPro palliative SOC cohort

Marker	FMI	digipath	scRNA	scDNA	CyTOF	IMC	Proteotype	Pharmacoscopy	4iDRP
BRAF	1								
MAP2K1	1		1	1			1		
NRAS (mutation)	1						0		
RAF1	1		1	1			1		
CTLA-4			1		1	1	1		
exhausted_CD8			1		1	1			
HLA-ABC			1		1	1	1		
IFN_gamma			1			1			
MAPK			1			1	1		1
PD-1		1	1		1	1			
PD-L1		1	1		1	1			
Perforin_CD8+			1			1			
TMB	1								
apoptosis			1			1			1
proliferation			1			1			1
pAKT			1			1			1
pEGFR						1			1
pER					1	1			1
pERK					1	1			1
pMET					1	1			1
chemotherapy								1	1
MEKi								1	1
MEKi+BRAFi								1	1
Interaction_CD8_PD1+_with_tumor_PD-L1+						1			
T_cell_infiltration		1				1			
TLS		1				1			

Supplementary Table M2b: Marker equivalencies table for the TuPro palliative beyond SOC cohort

Marker	FMI	digipath	scRNA	scDNA	CyTOF	IMC	Proteotype	Pharmacoscopy	4iDRP
BRAF	1								
CCND1	1		1	1			1		
CDK4	1		1	1	1	1	1		
CDK6	1		1	1	1	1	1		
CDKN2A/B	1		1	1		1	1		
EGFR	1		1	1	1	1	1		1
FGFR1	1		1	1	1	1	1		
KDR	1		1	1			1		
c-KIT	1		1	1	1	1	1		1
KIT	1								
MAP2K1	1		1	1			1		
MET	1		1	1	1	1	1		
NF1	1								
NRAS (mutati	1						0		
PDGFRA	1		1	1	1	1	1		
SRC	1		1	1			1		
E-Cadherin			1			1	1		
GNA11	1								
GNAQ	1								
HLA-ABC			1		1	1	1		
HRAS	1								
IFN_gamma+ T_cells			1			1			
MAPK			1			1	1		1
mTOR			1		1	1	1		1
PD-L1		1	1		1	1			
PRKC			1			1	1		
VEGF-A			1		1	1	1		
VEGF-B			1		1	1	1		
TMB	1								
apoptosis			1			1			1
proliferation			1			1			1
pAKT			1			1			1
pEGFR						1			1
pERK					1	1			1
pMET					1	1			1
TKI								1	1
BRAFi								1	1
chemotherapy								1	1
MEKi								1	1
MSI	1								
T_cell_ infiltration		1				1			
TLS		1				1			

Supplementary Table M3: Eight (=8) patients with serial biopsies (n=2 per patient). Shown are patient sample IDs for all eight patients, treatment recommendations, cohort classification and markers identified by various technologies applied with the TuPro project.

Patient	Sample ID	Treatment	Cohort	Markers
1	MEMEMUH	Nivolumab/PEG-IL2	beyond SOC	CyTOF: HLA-ABC PD-L1, IMC: HLA-ABC E-Cadherin, scRNA: E-Cadherin HLA-ABC CIFN_gamma_CD8+, Granzyme_B_CD8+ Perforin_CD8+
1	MUKAGOX	Binimetinib/Encorafenib	SOC	FMI: BRAF, CyTOF: pERK
2	MANOFYB	Ipilimumab/Nivolumab	SOC	FMI: TMB
2	MIGEKUT	Cisplatin/Vindesin	SOC	digipath: immune_excluded, IMC: immune_excluded
3	MEBIGAL	Ipilimumab/Nivolumab	SOC	FMI: TMB, digipath: inflamed
3	MUFOFOP	Dabrafenib/Trametinib	SOC	FMI: BRAF MAP2K1_(MEK1)
4	MODALEG	Ipilimumab/Nivolumab	SOC	FMI: TMB, IMC: HLA-ABC
4	MUCADOP	Ipilimumab/Nivolumab	SOC	FMI: TMB, digipath: TLS, scRNA: IFN_gamma CTLA-4, CyTOF: PD-L1 CTLA-4, IMC: PD-L1
5	MIMOHIS	Ipilimumab/Nivolumab	SOC	FMI: TMB, IMC: HLA-ABC, CyTOF: HLA-ABC
5	MUFYDUM	Ipilimumab/Nivolumab	SOC	FMI: TMB, IMC: HLA-ABC PD-L1
6	MADUFEM	Atezolizumab/Sunitinib	beyond SOC	FMI: KIT TMB, CyTOF: KIT VEGF-A, digipath: TLS PD-L1, IMC: PD-L1 HLA-ABC
6	MEWORAT	Carboplatin/Sunitinib/ Temozolomide	beyond SOC	FMI: KIT KDR PDGFRA
7	MEMIGOG	Nivolumab/Trametinib	beyond SOC	FMI: TMB BRAF HRAS NF1, CyTOF: PD-L1 HLA-ABC pERK, IMC: HLA-ABC pERK, Pharmacoscopy: MEKi
7	MIPYNAP	Nivolumab/Trametinib	beyond SOC	FMI: TMB BRAF HRAS NF1, Pharmacoscopy: MEKi, 4iDRP: pERK proliferation
8	MYKOKIG2	Ribociclib/Trametinib	beyond SOC	FMI: CCND1, scRNA: CCND1 CDK4 MAPK_pathway, CyTOF: CDK4 CDK6
8	MYNELIC1	Ribociclib/Trametinib	beyond SOC	FMI: CCND1, 4iDRP: pEGFR pERK, CyTOF: pERK CDK4 CDK6, scRNA: CCND1 MAPK_pathway

Supplementary Table M4: All drugs and drug combinations given in the Non-TuPro cohort.

Atezolizumab/Cobimetinib/Vemurafenib	Atezolizumab/Sunitinib	Bempegaldesleukin/Nivolumab	Binimetinib
Binimetinib/Dabrafenib/Encorafenib/Trametinib	Binimetinib/Encorafenib	Binimetinib/Encorafenib/Nivolumab	Binimetinib/Encorafenib/Nivolumab intrathecal
Binimetinib/Encorafenib/Pembrolizumab	Binimetinib/Nivolumab	Binimetinib/Paclitaxel	Binimetinib/Pembrolizumab
Cabozantinib/Nivolumab	Carboplatin	Carboplatin/Paclitaxel	Carboplatin/Paclitaxel/Sunitinib
Carboplatin/Sunitinib/Temozolomide	Carboplatin/Talimogene laherparepvec/Temozolomide	Carboplatin/Temozolomide	Carboplatin/Trametinib
Cisplatin/Temozolomide	Cisplatin/Vindesine	Cobimetinib	Cobimetinib/Erlotinib
Cobimetinib/Regorafenib	Cobimetinib/Temozolomide	Cobimetinib/Vemurafenib	Crizotinib
Dabrafenib/Ipilimumab/Nivolumab/Trametinib	Dabrafenib/Pembrolizumab/Trametinib	Dabrafenib/Regorafenib/Trametinib	Dabrafenib/Ribociclib/Trametinib
Dabrafenib/Spartalizumab/Trametinib	Dabrafenib/Trametinib	Dacarbazine	Dacarbazine/Palbociclib
Dacarbazine/Ribociclib	Dasatinib	Gebasaxturev/Pembrolizumab	Imatinib
Ipilimumab/Nivolumab	Ipilimumab/Nivolumab or Nivolumab or Ipilimumab	Ipilimumab/Nivolumab/Relatlimab	Ipilimumab/Nivolumab/Temozolomide
Ipilimumab/Trametinib/Nivolumab	Lenvatinib/Pembrolizumab/Quavonlimab	Linrodostat/Nivolumab/Relatlimab	Naporafenib/Ribociclib
Naporafenib/Spartalizumab	Nilotinib	Nivolumab	Nivolumab intrathecal
Nivolumab/Nivolumab intrathecal/Relatlimab	Nivolumab/Relatlimab	Nivolumab/Relatlimab/Ribociclib	Nivolumab/Ribociclib
Nivolumab/Temozolomide	Nivolumab/Trametinib	Nivolumab/Visugromab	Olaparib/Temozolomide
Paclitaxel	Paclitaxel/Ribociclib	Paclitaxel/Temozolomide	Pembrolizumab
Pembrolizumab/Lenvatinib or Placebo	Pembrolizumab/Quavonlimab	Pembrolizumab/Vibostolimab	Pemprolizumab/Trametinib
Regorafenib	Ribociclib/Spartalizumab	Ribociclib/Temozolomide/Vindesine	Ribociclib/Trametinib
Sunitinib	Talimogene laherparepvec	Tebentafusp	Temozolomide
Trametinib	Trametinib/Vindesine		
