# Supplementary material for: Feasibility of multiomics tumor profiling for guiding treatment of melanoma
Source: Nat Med. 2025 May 27;31(7):2430–41. doi: 10.1038/s41591-025-03715-6 (PMC12283375; doi:10.1038/s41591-025-03715-6)
Supplement: Supplementary file 2 — Reporting Summary [file 41591_2025_3715_MOESM2_ESM.pdf]

nature portfolio

Corresponding author(s): Andreas Wicki

Last updated by author(s): Apr 7, 2025

Reporting Summary

Nature Portfolio wishes to improve the reproducibility of the work that we publish. This form provides structure for consistency and transparency in reporting. For further information on Nature Portfolio policies, see our Editorial Policies and the Editorial Policy Checklist.

Statistics

For all statistical analyses, confirm that the following items are present in the figure legend, table legend, main text, or Methods section.

n/a	Confirmed	
☐	☒	The exact sample size (n) for each experimental group/condition, given as a discrete number and unit of measurement
☐	☒	A statement on whether measurements were taken from distinct samples or whether the same sample was measured repeatedly
☐	☒	The statistical test(s) used AND whether they are one- or two-sided<br>*Only common tests should be described solely by name; describe more complex techniques in the Methods section.*
☐	☒	A description of all covariates tested
☐	☒	A description of any assumptions or corrections, such as tests of normality and adjustment for multiple comparisons
☐	☒	A full description of the statistical parameters including central tendency (e.g. means) or other basic estimates (e.g. regression coefficient) AND variation (e.g. standard deviation) or associated estimates of uncertainty (e.g. confidence intervals)
☐	☒	For null hypothesis testing, the test statistic (e.g. F, t, r) with confidence intervals, effect sizes, degrees of freedom and P value noted<br>*Give P values as exact values whenever suitable.*
☒	☐	For Bayesian analysis, information on the choice of priors and Markov chain Monte Carlo settings
☒	☐	For hierarchical and complex designs, identification of the appropriate level for tests and full reporting of outcomes
☒	☐	Estimates of effect sizes (e.g. Cohen's d, Pearson's r), indicating how they were calculated

Our web collection on statistics for biologists contains articles on many of the points above.

Software and code

Policy information about availability of computer code

Data collection	Electronic data capture for the clinical data was conducted in secuTrial®, v6.1.2.5.
Data analysis	Data analysis, statistical testing and visualization were conducted in R (R Core Team, Version 4.4).<br>TuPro treatments were matched (1:1) to the non-TuPro treatments using a genetic matching algorithm (MatchIt package, Version 4.5.5).

For manuscripts utilizing custom algorithms or software that are central to the research but not yet described in published literature, software must be made available to editors and reviewers. We strongly encourage code deposition in a community repository (e.g. GitHub). See the Nature Portfolio guidelines for submitting code & software for further information.

Data

Policy information about availability of data

All manuscripts must include a data availability statement. This statement should provide the following information, where applicable:

- Accession codes, unique identifiers, or web links for publicly available datasets
- A description of any restrictions on data availability
- For clinical datasets or third party data, please ensure that the statement adheres to our policy

To comply with applicable laws and regulations (the Swiss Human Research Act), all de-identified clinical data relevant to this publication are provided as supporting information to the paper. Access to the patient-level clinical and biological data presented at the molecular tumor boards will be granted to registered users listed on the data access agreement with the Tumor Profiler Consortium (TPC) within four weeks of receipt of the Data Access Agreement, provided that the applicant

submits all necessary ethics committee approval and supporting documents needed to meet the requirements of the agreement. Data access can be requested by contacting the TPC (nicola.miglino@usz.ch). The user institution agrees to destroy or discard the data once it is no longer used for the project, and in cases where data must be archived, it must be deleted within 10 years of the project's completion. If data has not been archived, it must be deleted no later than 2 years following the completion of the project. An extension to this period can be provided upon request to the TPC leadership. Data sharing is subject to honoring patient privacy and data integrity.

Human research participants

Policy information about studies involving human research participants and Sex and Gender in Research.

Reporting on sex and gender	There was no sex or gender based analysis of data.
Population characteristics	Both female and male participants, age range 20-89. Distribution of driver mutation classes: RAF (40%), NRAS (25%), NF1 (5.5%), wild type (13.5%). Stage III or IV cutaneous melanoma, or rare melanoma subtypes at any stage that require systemic therapy: cutaneous (incl. acral) (77%), ocular (8.7%), melanoma of unknown primary (8.7%), mucosal melanoma (5.6%). ECOG performance status ≤2 (not bedridden for more than 50% of waking hours).
Recruitment	Screening of potential eligibile patients for this and other studies occured at the regular weakly tumor board. Eligible patients during routine clinical visits were informed and asked about their participation in the study. Patients meeting inclusion criteria were included after receiving detailed information on the study procedures and upon written informed consent according to the study-specific SOP for accrual. Study data were entered into SecuTrial and a study ID was generated. After routine biopsy as per clinical standard, left-over tissue material and a blood sample was sent to the central lab with assigned sample IDs. Participants did not receive compensation for their involvement in the study.
Ethics oversight	TuPro was conducted as a multi-center precision oncology project according to the Swiss Act and Ordinance on Human Research (HFG and HFV) with approval of regulatory authorities (ethics committees of northwestern Switzerland, EKNZ, and of Zurich, KEK). (EC-ID: 2018-02050, 2021-01584)

Note that full information on the approval of the study protocol must also be provided in the manuscript.

Field-specific reporting

Please select the one below that is the best fit for your research. If you are not sure, read the appropriate sections before making your selection.

☒ Life sciences ☐ Behavioural & social sciences ☐ Ecological, evolutionary & environmental sciences

For a reference copy of the document with all sections, see nature.com/documents/nr-reporting-summary-flat.pdf

Life sciences study design

All studies must disclose on these points even when the disclosure is negative.

Sample size	This study is an observational feasibility project without powered sample size.
Data exclusions	All collected data were included in the analysis, and no data were excluded at any stage.
Replication	This study is an observational feasibility project. Further randomized studies are necessary to ultimately ensure reproducibility.
Randomization	Due to the nature of this study as a feasibility project, no randomization was applied.
Blinding	Due to the nature of this study as a feasibility project, no blinding was applied.

Reporting for specific materials, systems and methods

We require information from authors about some types of materials, experimental systems and methods used in many studies. Here, indicate whether each material, system or method listed is relevant to your study. If you are not sure if a list item applies to your research, read the appropriate section before selecting a response.

Materials & experimental systems

n/a	Involved in the study
☒	☐ Antibodies
☒	☐ Eukaryotic cell lines
☒	☐ Palaeontology and archaeology
☒	☐ Animals and other organisms
☐	☒ Clinical data
☒	☐ Dual use research of concern

Methods

n/a	Involved in the study
☒	☐ ChIP-seq
☒	☐ Flow cytometry
☒	☐ MRI-based neuroimaging

Clinical data

Policy information about clinical studies

All manuscripts should comply with the ICMJE guidelines for publication of clinical research and a completed CONSORT checklist must be included with all submissions.

Clinical trial registration: NCT06463509

Study protocol: The full study protocol is available on request (data@tumorprofilercenter.ch).

Data collection: The project was conducted at the Department of Dermatology, University Hospital Zurich, the Department of Oncology and Hematology, Kantonsspital Baselland, and the Department of Oncology, University Hospital Basel, Switzerland, from January 2019 until November 2020 (BASEC: 2018-02050, 2021-01584) in accordance with the applicable legal and institutional standards.

Outcomes: TuPro aims to establish a platform for in-depth tumour profiling in patients with advanced melanoma. Goals of this platform are to establish logistics and algorithms for integrative analyses, discover new molecular biomarker profiles/patterns, and ultimately enable us to design and conduct future molecular matched clinical trials. As a feasibility project, outcomes were broadly defined as follows:

1. Feasibility outcomes:
-Types of molecular information and combinations of molecular information from the biotechnology domain that the pre-Tumour Board considers as useful for making a treatment recommendation beyond routine diagnostics (incl. routine pathology and NGS testing)

2. Classification of proposed treatment options based on Tupro measurements:
- On-label treatment with molecular matched treatment (SwissMedic label as reference) +/- radiotherapy or chemotherapy;
- Treatment with classical chemotherapy +/- radiotherapy (on label if label available);
- Referral to a suitable clinical trial;
- Off-label treatment (SwissMedic label as reference) with molecular matched treat-ment or immunotherapy +/- radiotherapy or chemotherapy;
- Off-label treatment (authorization in countries with comparable control systems for medicinal products as defined by SwissMedic) with molecular matched treatment or immunotherapy +/- radiotherapy or chemotherapy;
- Immunotherapy
- No active anti-tumour treatment (best supportive care).

3. Clinical outcomes
- Best response to treatment, assessed radiologically after treatment initiation, classified according to RECIST criteria.
- Progression-free survival as the duration in months between the date of treatment initiation (first medication intake or application) and the date of the radiologically confirmed progression, if progression occurred.
